# Three-Dimensionally Printed Paediatric Medicines: Formulation, Process, and Regulatory Considerations

**DOI:** 10.3390/pharmaceutics18010002

**Published:** 2025-12-19

**Authors:** Krisztina Petrinca, Zsófia Németh, Ildikó Csóka, Rita Ambrus, Orsolya Jójárt-Laczkovich

**Affiliations:** Institute of Pharmaceutical Technology and Regulatory Affairs, University of Szeged, Eötvös Street 6, 6720 Szeged, Hungary; petrinca.krisztina@szte.hu (K.P.); nemeth.zsofia@szte.hu (Z.N.); csoka.ildiko@szte.hu (I.C.); jojartne.laczkovich.orsolya@szte.hu (O.J.-L.)

**Keywords:** paediatrics, 3D printing, individualised therapy, paediatric dosage forms, 3D-printed medicine

## Abstract

Paediatric formulations are pharmaceutical dosage forms specifically designed to meet the physiological, developmental, pharmacokinetic, and practical needs of patients from birth to adolescence. Developing safe, effective, and age-appropriate medicines for children remains a significant challenge due to their age-dependent variability in physiological development, pharmacokinetic profiles, and therapeutic needs. These differences, combined with practical barriers such as poor palatability, limited swallowability, inappropriate dosage form size, and instability, often lead to the modification of adult medicines—practices that can cause dosing inaccuracies, contamination risks, and reduced therapeutic efficacy. Three-dimensional printing has emerged as a promising solution to address these limitations by creating personalised paediatric dosage forms with adjustable strengths, multilayer structures for controlled release, and child-friendly shapes that may improve acceptability and adherence. This review offers an overview of the physiological, technological, and regulatory factors involved in developing 3D-printed paediatric medicines. The Critical Quality and Performance Attributes relevant to this field—including dose accuracy and flexibility, release kinetics, palatability, product dimensions, material choice, safety, stability, cost-effectiveness, production time, scalability, and reproducibility—are discussed in the article. Additionally, the review discusses the evolving Good Manufacturing Practice and regulatory landscape necessary to ensure the quality, safety, and consistency of 3D-printed medicinal products. Overall, these insights underline the transformative potential of 3D printing as a pathway towards safer, more effective, and truly personalised pharmacotherapy for paediatric patients.

## 1. Introduction

Paediatrics is the branch of medicine that deals with the diseases and treatment of children [1].

Providing ill children with the most suitable and preferred medicine to recover as soon as possible would be the best solution, but it is not always feasible; most of the time, it is a complex and challenging task. Paediatric formulation development is driven by the need to design therapeutic dosage forms that address the unique physiological characteristics and requirements of children. [2,3]

Children form a heterogeneous group, as their physical development, as well as their pharmacokinetic and pharmacodynamic characteristics, vary with age [4]. The International Conference on Harmonisation of Technical Requirements for Registration of Pharmaceuticals for Human Use (ICH) classified age groups from birth to adulthood in the following way:

- Preterm newborn infants;
- Term newborn infants (0–27 days);
- Infants and toddlers (28 days–23 months);
- Children (2–11 years);
- Adolescents (12 to 16–18 years (dependent on region)) [5].

Paediatric diversity is present not only in terms of size and age groups, but also in the unique pharmaceutical needs. From birth to adulthood, children undergo numerous changes in pharmacokinetics and pharmacodynamics, including differences in drug metabolism, absorption, distribution, and elimination. These variabilities are due to age-dependent variations in organ function, enzyme activity, gastric pH, intestinal permeability, and renal clearance [6]. For example, preterm newborn infants have different needs than term newborn infants; they require different doses of a given active ingredient and may be more susceptible to illnesses that do not occur among term newborn infants [7].

Ensuring that formulations are age-appropriate, safe, and effective is a central regulatory requirement in paediatric drug development. Consequently, agencies such as the European Medicines Agency (EMA), the U.S. Food and Drug Administration (FDA), and the World Health Organisation (WHO) emphasise the need for paediatric-specific dosage forms that account for developmental pharmacokinetics and pharmacodynamics (PK/PD). Despite regulatory progress, the use of off-label and unlicensed medicines in children remains common. Although often perceived as an issue of the past, multiple studies demonstrate that it persists. For example, Berkovich et al. compared data from 2002 and 2018 and found that off-label and unlicensed medicines are still widely used in clinical practice [8,9]. The absence of appropriately designed paediatric formulations contributes substantially to this problem. As a result, adult medications are frequently split, crushed, or otherwise modified for paediatric use, increasing the risk of inaccurate dosing, poor therapeutic efficacy, or toxicity, resulting in instability in stored liquid preparations and uneven subdivision of solid forms [6,10].

Although several drug formulations can be suitable for children, the optimal approach is personalised therapy. For this reason, medicines are often modified to create ‘extemporaneous’ formulations, which are freshly prepared pharmaceuticals tailored to individualise the dosage of a medicinal product or accommodate other specific patient needs, such as sensory processing disorders, food allergies, or dietary restrictions [11]. However, while such manipulations may save lives, their safety and effectiveness have not been established through systematic quality control [12].

Compounding remains common in paediatric practice due to the lack of age-appropriate dosage forms, yet it carries risks such as contamination and dosing errors; it also requires highly trained personnel and specialised facilities that are not universally available [11]. Recent data indicate that since 2019, 3.0% of prescriptions dispensed by Dutch community pharmacies required manipulation, while this proportion reached 10.5% at the Erasmus MC Sophia Children’s Hospital [13]. The most frequently manipulated medications in Dutch community pharmacies were lactulose syrup, oxazepam, dipiperone, haloperidol, prednisolone, lorazepam, mirtazapine, levothyroxine, amoxicillin, and valproic acid, while among children the most commonly manipulated APIs were amoxicillin, desloratadine, methylphenidate, nystatin, amoxicillin/clavulanic acid, omeprazole, azithromycin, cotrimoxazole, nitrofurantoin, and ranitidine [13]. Population-level drug-use patterns aligned with national prescribing data, suggesting that these findings may be generalisable to the broader Dutch population and potentially extendable to other European countries, while considering regional differences in drug availability and prescribing behaviour, e.g., variations in antibiotic usage across Europe [13].

As dose adjustment remains a challenge in paediatrics, three-dimensional printing (3DP) has emerged as a promising technology for producing precise, subdivided, and personalised doses that outperform conventional compounding in terms of accuracy and material efficiency [14]. Three-dimensional printing enables the manufacture of customised dosage forms without the loss of active pharmaceutical ingredient (API), supporting both personalised therapy and cost-effective use of raw materials [10]. According to Pannekoek et al., the APIs most suitable for 3DP-enabled compounding are those currently manipulated by patients or healthcare professionals, such as psychopharmaceuticals, cardiovascular, and anti-infective drugs, because manual handling introduces clinically significant risks of their dosing inaccuracy [13]. Overall, 3DP presents a viable future strategy for reducing manipulation-related risks and enhancing the safety and suitability of paediatric medicines [13,14].

This article aims to provide a thorough overview of the key considerations involved in developing medicines for the paediatric population, with particular emphasis on physiological, pharmacokinetic, and practical factors that influence formulation design. A further objective is to assess the potential of three-dimensional (3D) printing in producing safe, accurate, and personalised medicines for this patient group. To achieve this, the review considers the quality standards for paediatric formulations and discusses the Good Manufacturing Practice (GMP) framework as it relates to the production of 3D-printed medicinal products. Collectively, these objectives aim to illustrate how 3D printing could provide a feasible pathway to enhance the quality, safety, and appropriateness of paediatric pharmacotherapy.

## 2. Legislative Considerations Regarding Paediatric Medicine

Throughout history, several actions have led to restrictions in the pharmaceutical industry. In 1906, the American Pure Food and Drug Act prohibited the sale of misbranded or adulterated food and drugs, laying the foundation for the nation’s first consumer protection agency, the FDA [15,16]. Many products slipped through this act, and later, in 1937, a new antibiotic, Elixir Sulphanilamide, caused the death of more than one hundred people, primarily children. Later, it was discovered that the antibiotic contained a toxic chemical compound due to a lack of safety testing, as it had been investigated only for its flavour, appearance, and fragrance. This action led to the enactment of the Food, Drug and Cosmetic (FD&C) Act of 1938 [15,17].

A few years later, thalidomide was marketed as a good sleeping pill by the German firm Chemie Grunenthal, recommended for pregnant women for morning sickness as well. As a result, in Western Europe, thousands of babies were born with malformation. In response to this news, in 1962, the US Congress passed the Kefauver-Harris Drug Amendments to the Federal Food, Drug, and Cosmetic Act, which require firms to demonstrate not only safety but also effectiveness for the intended use of the product [18].

These tragedies led to the development of a legislative framework for pharmaceuticals.

### 2.1. Worldwide Alliance

Although each continent and country has its own regulations, there is a significant need for an organisation that connects agencies worldwide; this organisation is the WHO. The WHO was founded in 1948 to connect nations, save the vulnerable, and improve healthcare globally. This date is now celebrated as World Health Day yearly [19]. The WHO works to address global health issues by bringing together 194 countries. Their goal is to provide every person in the world with an equal quality of life from birth to death [20]. The organisation gives advice in the form of guidelines, promotes research in poor countries, and advocates for children’s health and ethical research conduct: ‘Health promotion, disease prevention services (such as vaccinations) and treatment of common childhood illnesses are essential if children are to thrive as well as survive’ [21].

Global and regional data are available on the WHO website regarding children [22]. Based on their statistics, more mothers and children survive today than ever before. Yet there still are global health issues that claim the lives of children under the age of five, most of which could be prevented or treated if regular healthcare or decent quality of life were available in these regions. Malnutrition and environmental problems also impact the physical and mental development of young children [22].

Table 1 summarises the number of under-five deaths and their causes in the years 2020 and 2021 (the latest data available on the WHO website as of 2025) [23]. Premature birth, acute lower respiratory infections, birth asphyxia and trauma are the leading causes of death in this age group, drawing attention to the vulnerability at birth and early ages.

### 2.2. American Regulations

Regulations in the United States of America (USA) have a significant impact on pharmaceutical development, not only for adults but also for paediatric drug formulations and indications. Without these regulations, fewer medicines would be available for the paediatric population due to the lack of incentives and motivation the government has provided to industry.

The first developments in paediatric legislation began in the USA on 21 November 1997, with the enactment of the Food and Drug Administration Modernisation Act (FDAMA). This legislation focuses on reforming the regulation of food, medical products, and cosmetics. The act has 7 fundamental provisions:

- Prescription drug user fees;
- FDA initiatives and programmes;
- Information on off-label use and drug economics;
- Pharmacy compounding;
- Risk-based regulation of medical devices;
- Food safety and labelling;
- Standards for medical products [24].

In the following year, the Paediatric Rule of 1998 was enacted, which mandated sponsors to include paediatric assessments of safety and efficacy in applications for new drug or biologic therapies, new indications, new dosage forms, new dosing regimens, or new routes of administration for existing therapies that are likely to be used in paediatric patients. However, the Paediatric Rule, which firmly based the paediatric regulations in the USA, was invalidated in 2002 after concluding that its requirement for pharmaceutical companies to test the safety of adult medicines in children is contrary to the intent of Congress [25].

The Best Pharmaceutical for Children Act (BPCA) is part of the FDAMA of 1997, but became law only in 2002. This regulation encourages the pharmaceutical industry to perform paediatric studies to improve labelling for patented drug products used in children; also, to authorise the National Institutes of Health (NIH) to prioritise needs in various therapeutic areas and sponsor clinical trials of off-patent drug products that need further study in children, as well as training and other research that addresses knowledge gaps in paediatric therapeutics. The NIH’s BPCA programme requires renewal every five years; the most recent renewal occurred in 2022 [26].

A year later, in 2003, the Paediatric Research Equity Act (PREA) was enacted to authorise the FDA to require paediatric studies to define the best route of administration, the correct dosage, dosage form, and indication. It applies not only to the new but also to the already marketed APIs. The pharmaceutical industry has the right to refuse these investigations if their new drug is not intended to be applied to children. PREA excludes cancer drugs and orphan drugs, which treat rare conditions that do not occur among children [27].

**Figure 1:**
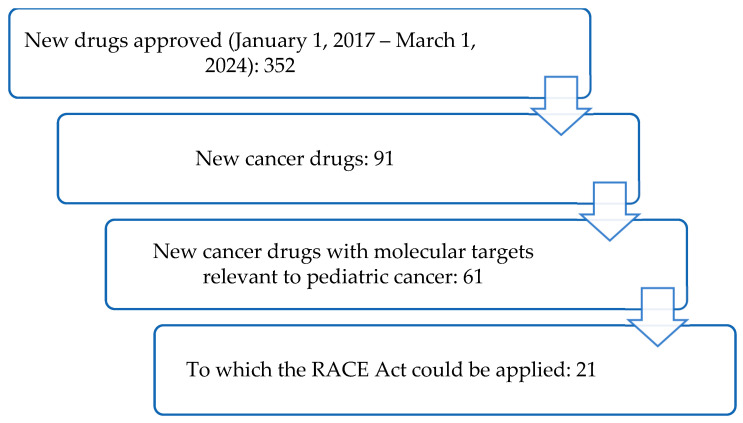
Flow diagram of new, molecularly targeted adult cancer drug approvals between 2021 and 2024 [28].

The Congress enacted the Research to Accelerate Cures and Equity (RACE) for Children Act in 2017, which expands treatment options for paediatric cancer patients by authorising that any new oncology drugs, even those initially intended to cure adult cancers only, must also be tested on children. This law took effect on 18th August 2020. Ian T. T. Liu et al. compiled all publicly available FDA data to compare paediatric post-approval requirements, trials, and trial characteristics, including timing, of adult cancer drugs before and after the RACE Act. Based on the collected data, between 2017 and 2024, the FDA approved 61 adult cancer drugs with molecular targets relevant to paediatric cancer; 40 were submitted before 2020, and 21 were submitted after 2020 out of the 352 approved medications during this period (Figure 1) [28].

The FDA’s Priority Review Voucher (PRV) programme provides a new incentive for pharmaceutical companies to develop more medications intended for children. Vouchers are granted to companies that successfully develop treatments for specific neglected diseases, such as rare paediatric diseases and tropical diseases, as well as certain medical countermeasures. The sponsor can use it to receive priority review of any subsequent drug application, which reduces the FDA review time from 10 months to 6 months. These vouchers can also be sold to other companies, creating a market and acting as a financial incentive to develop drugs for these otherwise less profitable areas. The PRVs can be transferred an unlimited number of times. There are three types of vouchers: Tropical Disease Vouchers, Rare Paediatric Disease PRVs, and Material Threat Medical Countermeasure PRVs. The company using a PRV must notify the FDA at least 90 days before its application [29].

The Tropical Disease PRV programme was first authorised in 2007 to encourage the pharmaceutical industry to develop treatments for tropical diseases, such as tuberculosis and malaria. In 2014, Ebola was added to the list [30].

The Rare Paediatric Disease PRV programme is an incentive programme for paediatric studies, specifically for sponsors who do not seek an adult indication and evaluate a rare disease in children. According to the Advancing Hope Act of 2016, a disease can be considered a rare paediatric disease if “the disease is a serious or life-threatening disease in which the serious or life-threatening manifestations primarily affect individuals aged from birth to 18 years, including age groups often called neonates, infants, children, and adolescents” [30]. The Rare Paediatric Disease PRV programme was established in 2007 and enacted in 2012 under the 2012 Food and Drug Administration Safety and Innovation Act (FDASIA), and reauthorised under the 2016 Advancing Hope Act. The programme is designed to spur the development of new treatments for diseases that would otherwise not attract company interest due to high costs and limited market opportunities, including tumour therapy. The future of this programme is uncertain; it is set to expire, and while legislative efforts to reauthorise it are underway, a new law has not yet been passed. The designation deadline for the programme was 20 December 2024; a drug must have received its rare paediatric disease designation by this date and be approved by 30 September 2026, to be eligible for a voucher [31].

The Material Threat Medical Countermeasure PRV programme was established in 2016, as mandated by the 21st Century Cures Act. It was established to encourage the development of medical countermeasures by offering incentives to investigate harmful biological, chemical, radiological, or nuclear materials during the development of new drugs or biological medical products [29].

Table 2 summarises the main information about these vouchers.

### 2.3. European Regulations

In the European Union, the EMA coordinates the evaluation, supervision, and safety monitoring of medicines [32]. The EMA was founded in 1995 and is responsible for ensuring the safe and efficient development, manufacture, and supply of human and veterinary medicines in the European Union, Iceland, Norway, and Liechtenstein [33]. With its seven committees—Pharmacovigilance Risk Assessment Committee (PRAC), Committee for Orphan Medicinal Products (COMP), Committee for Advanced Therapies (CAT), Committee for Medicinal Products for Human Use (CHMP), Committee for Veterinary Medicinal Products (CVMP), Committee on Herbal Medicinal Products (HMPC), Paediatric Committee (PDCO)—the Agency can conduct a qualitative scientific work, harmonise the national regulations, and plays essential role in clinical trial regulations [34]. Regarding the EMA’s database, there are 44,341 clinical trials performed with EudraCT protocol, of which 7370 are trials conducted with children (subjects younger than 18 years) [35].

**Figure 2:**
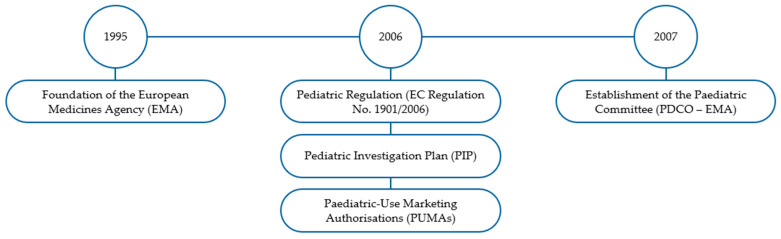
Graphical summary of the European legislative milestones.

Paediatric legislation in Europe has evolved significantly over the past two decades (see Figure 2), primarily driven by the need to ensure that children have access to safe and effective medicines.

The cornerstone of this legislative framework is Regulation (EC) No. 1901/2006, commonly referred to as the Paediatric Regulation. This regulation was implemented to enhance the development of medicines specifically for children and adolescents [36,37,38]. The regulation has significantly transformed the landscape of paediatric drug development in Europe by mandating the inclusion of paediatric considerations in the drug approval process. Similarly to the results in the USA, the regulation has led to an increase in the number of medicines authorised for use in children.

The Paediatric Regulation requires pharmaceutical companies to submit a Paediatric Investigation Plan (PIP) as part of their marketing authorisation application for new drugs, outlining the planned studies to assess the efficacy and safety of the drug in paediatric populations [39,40]. The EMA plays a crucial role in implementing the Paediatric Regulation. In 2007, the establishment of the Paediatric Committee (PDCO) within the EMA facilitated the evaluation of PIPs and the overall paediatric drug development process [41]. Since the Paediatric Regulation came into force, the number of clinical trials investigating medicinal products in paediatric populations has increased, accompanied by a corresponding rise in new marketing authorisations for paediatric medicines in the EU [42,43,44]. The EMA’s efforts have led to a more structured approach to paediatric drug development, ensuring that the specific needs of children are considered during the drug approval process [41]. PIPs are essential for the development of paediatric medicines, as they outline the studies that must be conducted in children. The regulation stipulates that PIPs must be submitted alongside the marketing authorisation applications for new drugs, ensuring that paediatric considerations are integrated into the dossier [45].

On the other hand, Paediatric-Use Marketing Authorisations (PUMAs) provide a pathway for medicines developed specifically for children, allowing for a streamlined approval process [46].

Despite the advancements brought about by the Paediatric Regulation, several challenges persist in the development of paediatric formulations:

- Off-label use: A meta-analysis indicated that globally, nearly 50% of the paediatric prescriptions were about either unlicensed or off-label medications [47], meaning they have not been specifically studied or approved for paediatric application and can lead to drug misuse, overdosage or adverse drug reactions [48,49].
- Formulation issues: Many existing formulations are not suitable for children due to their taste, size, or dosage form. This may lead to difficulties in adherence and therapeutic failure [7,50].
- Data availability: There is ongoing debate about the appropriateness of extrapolating data from adult studies to paediatric populations, given the physiological differences between these groups. The pharmaceutical industry must ensure that paediatric studies are designed ethically and that the rights of child participants are protected, which is paramount [51].
- Ethical considerations: The vulnerability of children as research subjects necessitates stringent ethical considerations, particularly regarding informed consent and the potential risks associated with clinical trials [52].
- Market incentives: The economic viability of developing paediatric formulations is often questioned, as the market for paediatric medications is smaller compared to adult medicines, leading to fewer investments in research and development [53,54].

To address these challenges, several strategies can be implemented:

- Enhanced collaboration: Increased collaboration between pharmaceutical companies, regulatory bodies, and healthcare providers can facilitate the development of more suitable paediatric formulations [55].
- Regulatory support: Continued support from regulatory agencies, including incentives for developing paediatric formulations, can encourage pharmaceutical companies to invest in this area [56,57].
- Innovative formulation technologies: The use of novel formulation technologies, such as nanotechnology and 3D printing, may offer new avenues for creating child-friendly formulations.

As the landscape of paediatric medicine evolves with innovations, the ongoing dialogue among stakeholders, including regulatory bodies, pharmaceutical companies, and healthcare providers, will be essential to address these challenges and ensure that the therapeutic needs of children are met.

### 2.4. Three-Dimensional Printing Legislation

Over the last few years, 3D printing technology (3DP) has garnered significant attention across various industrial sectors, including healthcare. Using 3DP, there is infinite potential to create new tools, medical devices, accessories, and even individually designed medicines. A broad legal framework has been established to control this possibility [58].

In 2014, the European Commission published the ‘Overview of 3D printing & intellectual property law’ (Annex 3), which summarises the main points and regulations of 3D printing technology. It mentions 11 regulations that 3DP legislation is based on:

- Berne Convention [59];
- Agreement on Trade Related Aspects of Intellectual Property Rights [60];
- WIPO Copyright Treaty [61];
- Directive 98/71/EC of the European Parliament and of the Council of 13 October 1998 on the legal protection of designs [62];
- Directive 2000/31/EC of the European Parliament and of the Council of 8 June 2000 on certain legal aspects of information society services, in particular electronic commerce, in the Internal Market [63];
- Directive 2001/29/EC of the European Parliament and of the Council of 22 May 2001 on the harmonisation of certain aspects of copyright and related rights in the information society [64];
- Regulation No. 6/2002 of 12 December 2001 on Community designs [65];
- Directive 2004/48/EC of the European Parliament and of the Council of 29 April 2004 on the enforcement of intellectual property rights [66];
- Directive No. 2008/95/EC of 22 October 2008 to approximate the laws of the Member States relating to trade mark [67];
- Regulation No. 207/2009 of 26 February 2009 on the Community trade mark [68];
- Directive 2009/24/EC of the European Parliament and of the Council of 23 April 2009 on the legal protection of computer programmes [69,70].

The regulations mentioned above all focus on creators’ rights. These allow the authors to decide how their art can be used and by whom. Although there are several regulations, none of them can give 100% protection against copying the original products. Copyright law protects Computer-Aided Designs (CADs), but it cannot control the manufacturing of unauthorised brand replicas. This is the weak point of 3DP legislation, where development is really needed [58].

Although regulatory interest in additive manufacturing is rapidly growing, no major authority has yet established specific regulations for 3D-printed medicinal products. Currently, 3D-printed drugs are assessed under existing pharmaceutical frameworks, as neither the FDA nor the EMA has issued dedicated guidance for 3D-printed pharmaceuticals. Only 3D-printed medical devices are covered by formal documents, such as the FDA’s Additive Manufacturing Guidance, issued in 2018 [71]. As a result, issues like batch definition, point-of-care manufacturing, process validation, and quality control continue to be governed by traditional GMP-based expectations, which were designed for large-scale, centralised production rather than personalised, small-batch printing. This regulatory gap highlights the need for new, technology-specific guidance to support the safe integration of 3D-printed medicines into clinical practice, especially for paediatric use.

## 3. Biopharmaceutical Paediatric Considerations

Children form a diverse group, and they cannot be treated as small adults. Their biological and pharmaceutical properties differ from those of a fully grown human, necessitating specialised treatments and drug formulations with individualised, accurate dosages [72]. Understanding the inner environment of the diverse children group is challenging, despite several attempts to model their ADME (A = Absorption, D = Distribution, M = Metabolism, E = Excretion) characteristics [73].

**Figure 3:**
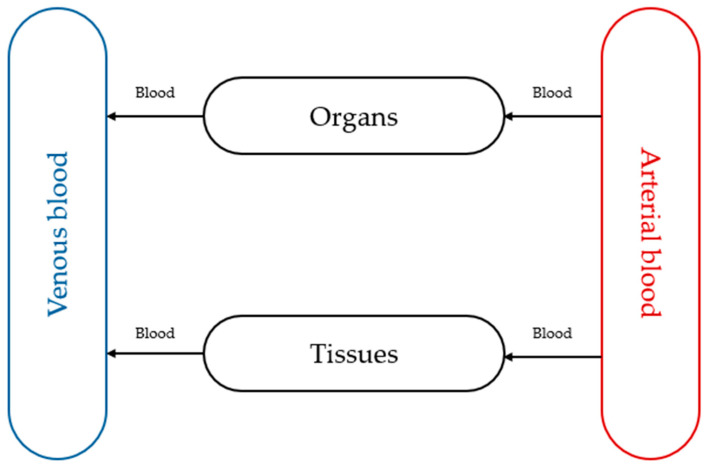
Schematic figure of a physiologically based pharmacokinetic (PBPK) model.

The paediatric physiologically based pharmacokinetic (PBPK) model integrates drug property data for children with developmental physiology. It is a mathematical approach used to predict and simulate how drugs behave in the developing bodies of children of different age groups, from neonates to adolescents. This method leverages existing adult data and incorporates age-dependent physiological changes to inform safe and effective paediatric drug dosing and development. The model has four main parts: venous blood circulation, arterial blood circulation, organs, and tissues. The structure of the PBPK model can be modified at any time based on the research purpose or type of API [74]. By changing demographic data (age, genetics, ethnicity, etc.), disease-specific data (the impact of the disease on pharmacokinetic properties, such as AUC, t_1/2_, or c_max_), and experimental factors (dosage form, food intake, and pH), various conditions can be analysed. This change enables simulating the appropriate ADME characteristics of a given population, such as children. Figure 3 shows the scheme of the PBPK model.

Due to physiological changes, infants and toddlers undergo rapid biopharmaceutical development during the first months of life. These developments start in the mother’s womb and last until the end of life. Although only changing is permanent, adults share similar pharmaceutical properties, which makes drug development for that age group less challenging [75]. In the following paragraphs, the pharmaceutical properties of paediatric and adult populations are compared to illustrate the challenges that must be faced when developing child-suitable medicines.

During pregnancy, the foetus gets nutrients (oxygen, vitamins, minerals, water, carbohydrates, etc.) from the mother through the placenta. This organ is also responsible for removing waste products (such as carbon dioxide) and protecting the foetus against infections, xenobiotics, and maternal diseases; it also produces hormones that provide adequate conditions and support the functions of both the mother and the foetus [76]. Although the placenta plays a significant role in reducing the exposure of the foetus to xenobiotics, many molecules, even bearing teratogenic effects (such as isotretinoin, thalidomide, alcohol, etc.), can cross it and affect foetal development [77].

After birth, the body weight and body surface area change rapidly, making individual therapy more challenging. For dose calculation, evidence suggests using the allometric scale:

(1) $$P_{\mathrm{child}}\;=\;P_{\mathrm{adult}}\;\cdot \;({\frac{WT}{70})}^{x}$$

where P is the parameter wished to be calculated, *WT* is the bodyweight of the individual, 70 is the average adult body weight, and *x* is the allometric exponent [78].

Although it is possible to calculate the optimal dose for each API, several biopharmaceutical factors must be considered during the determination process.

### 3.1. Absorption

Absorption depends primarily on pH, but intestinal transit time also plays a role.

Gastric pH is initially neutral at birth due to amniotic fluid filling the stomach; however, it typically drops to pH 3 within 1 or 2 days. By approximately 2 years of age, gastric pH decreases to around 1–2, reaching values comparable to those of adults [79]. Before this age, the relatively higher gastric pH can increase the oral bioavailability of acid-labile drugs, such as penicillin or erythromycin, because they undergo less degradation in the stomach. In contrast, the absorption of drugs whose solubility is enhanced in acidic conditions may be reduced in infants. This applies to weakly acidic compounds with pH-dependent solubility profiles, such as phenytoin, phenobarbital, or certain non-steroidal anti-inflammatory drugs. These developmental differences can influence systemic exposure and duration of action, and therefore must be considered when selecting the appropriate dose of the active pharmaceutical ingredient [80]. The gastric pH of preterm newborn infants, whose physiological development is not complete at birth, is even higher after 24 hours, approaching pH 4, which means the level of acid-labile drugs is higher than in term newborn infants due to a more alkaline gastric pH and worse renal function [81].

In young children, intestinal transit time is generally shorter than in older children or adults, which can reduce the absorption of drugs with slow dissolution or limited permeability; and more variability can be observed in particular transits, e.g., small intestinal transit is often faster in infants and toddlers, while colonic transit is often slower in infants [81].

The skin of children also differs significantly from that of adults. The stratum corneum is thinner, and skin hydration is higher during the first 6 months of life. Hydration then gradually decreases, reaching adult levels by approximately 1 to 2 years of age. These characteristics increase skin permeability and enhance chemical penetration, reflecting a reduced barrier function. As a consequence, transdermally or topically applied active ingredients may reach higher systemic concentrations, and inadequate dosing can lead to intoxication [82].

Intramuscular injections are a great way to administer several APIs; however, children have less muscle tissue, slower blood flow to muscles, and weaker muscle contractions. Together, these factors contribute to more variable and unpredictable drug absorption [83].

In several acute situations—such as nausea, seizures, or other emergencies—rectal administration can offer an effective and practical alternative. However, despite its advantages, suppositories and other rectal dosage forms (including liquids and gels) may irritate the rectal mucosa, depending on their excipients, pH, or osmolarity [84]. Additionally, due to individual differences in rectal contents, pH, or the rectal mucosal surface, absorption can be unpredictable [85].

### 3.2. Distribution

After absorption, drug molecules are distributed throughout the body. Distribution depends on several physiological factors, including total body water and extracellular water content, the proportion of adipose tissue, plasma protein-binding capacity, and membrane permeability [86].

Shortly after birth, total body water is markedly higher (80–90% of body weight) compared with adults (55–60%). Extracellular water can also be up to 20% higher in neonates and young children than in adults. These differences significantly affect the distribution of water-soluble drugs, as these compounds distribute into a larger aqueous volume, resulting in lower plasma concentrations at equivalent doses, e.g., gentamicin exhibits a larger volume of distribution in neonates [86].

Adipose tissue in children is thinner; therefore, lipophilic drugs, e.g., flunitrazepam, have a smaller volume to distribute in. As a result, the plasma concentrations of these molecules may be higher at equivalent doses [87].

Pharmakons with high protein-binding affinity may reach higher free plasma concentrations in children because plasma protein levels—especially albumin and α-1-acid glycoprotein—are lower than in adults. This increase in the unbound fraction can enhance pharmacological activity and raise the risk of toxicity, as seen with highly protein-bound drugs such as furosemide, phenytoin, and diazepam [88].

Drug molecules must also cross various biological membranes during distribution. In preterm and term newborns, the blood–brain barrier is not yet fully mature, which allows greater penetration of certain active substances and increases the risk of central nervous system toxicity. This is particularly relevant for sedating H1-receptor antagonists such as diphenhydramine or hydroxyzine, where infants may experience adverse effects—including convulsions, irritability, nervousness, or hyperactivity—more frequently than older children or adults [89,90].

### 3.3. Metabolism

Lipophilic drugs are poorly eliminated in their unchanged form; thus, they must first be metabolised to more hydrophilic metabolites to enable efficient renal excretion. The liver is the primary organ responsible for this biotransformation, although extrahepatic tissues, including the intestines, kidneys, lungs, and brain, also contribute to metabolic processes [86].

Drug metabolism processes can be categorised into two groups: phase I and phase II reactions, based on the underlying chemical backgrounds.

Phase I reactions involve adding reactive, polar functional groups to the central molecule to be excreted, thereby increasing its polarity and preparing it for subsequent Phase II conjugation. Cytochrome (CYP) P450 enzymes play a dominant role in these oxidative pathways [91]. In the prenatal period, the liver of the foetus produces mainly the CYP3A7 enzyme. Later in the postnatal period, CYP3A7 levels decline rapidly and disappear within a few weeks. In contrast, other CYP isoforms—such as CYP3A4, CYP2D6, and CYP2E1—gradually increase in activity during infancy, although their maturation follows non-linear and isoform-specific patterns. CYP1A2 is produced only 1–3 months after birth. As CYP1A2 is the primary enzyme responsible for caffeine metabolism, neonates rely instead on CYP2E1 and CYP3A-mediated pathways, resulting in prolonged caffeine elimination (half-life is 3–4 days, compared with approximately 5 hours in adults) [92,93].

Phase II reactions involve conjugation processes, in which endogenous molecules are added to drugs or their Phase I metabolites to increase their hydrophilicity and facilitate excretion. Key enzymes participating in these pathways include Uridine 5’-diphospho-(UDP)-glucuronosyltransferases (UGTs), sulfotransferases (SULTs), glutathione-S-transferases (GSTs), N-acetyltransferases (NATs), and thiopurine S-methyltransferase (TPMT). The grey-baby syndrome is a classic example of immature Phase II metabolism, resulting from reduced UGT activity. In the 1960s and 1970s, neonates treated with chloramphenicol developed severe toxicity because they were unable to glucuronidate and eliminate the drug adequately. The accumulation of the antibiotic led to cardiovascular collapse, abdominal distension, cyanosis, and, in many cases, death [86,94].

### 3.4. Excretion

Both glomerular filtration rate (GFR) and tubular secretion are reduced in infants and toddlers. As a result, drugs that are primarily eliminated renally, e.g., antibiotics, have a prolonged half-life in this age group [95,96].

Kidney function is not fully developed at birth; GFR rises rapidly during the first weeks of life and approaches adult values between approximately 1 and 2 years of age. Its maturation follows a sigmoid hyperbolic pattern, as described by den Bakker et al. [97].

The urine of young children is generally more acidic than that of adults, which enhances the reabsorption of weak acids and reduces the reabsorption of weak bases [98].

Table 3 summarises the main biopharmaceutical properties of children across different age groups.

### 3.5. Personalised Medicine

Children differ from adults not only in size but also in developmental pharmacology, and even within paediatric subgroups, considerable physiological variation exists. Personalised medicine aims to adapt therapy to the unique characteristics and needs of each patient, often involving the preparation of customised formulations through compounding. This approach is especially relevant in paediatrics, where age-dependent physiological variation and treatment adherence strongly influence therapeutic outcomes [3]. Customised formulations aim to enhance safety, efficacy, and acceptability by addressing swallowing difficulties, palatability issues, and the challenges associated with adapting adult dosage forms for children [10].

Although personalised therapy would significantly improve paediatric care, achieving it with conventional dosage forms remains challenging. Three-dimensional printing has therefore emerged as a promising tool of precision and personalised pharmacotherapy, allowing drug products to be designed according to age, weight, disease characteristics, or even genetic factors [2]. Its flexibility allows the production of complex pharmaceutical forms, including polypills that combine multiple APIs, products with modified release characteristics, and visually appealing child-friendly shapes, colours, or flavours, which can strengthen adherence and mitigate dosing errors [10,99]. Moreover, 3DP enables decentralised, on-demand manufacturing in hospitals and pharmacies, offering precise, personalised medicines for vulnerable groups, including paediatric and geriatric patients [99,100]. Furthermore, 3DP supports innovations that are not achievable with traditional compounding, such as incorporating Braille writing, QR codes, or other identifiers, and designing sophisticated geometries that allow for staged or delayed drug release [99,100]. Collectively, these capabilities highlight 3DP as a transformative technology for advancing highly individualised and patient-centred medicine [101,102,103,104].

## 4. Paediatric Medications

The paediatric population comprises more than 2.4 billion minors globally, including over 650 million children under five [92]. Despite children representing more than 20% of the European population—approximately 100 million individuals—over 70% of marketed medicines lack paediatric authorisation or adequate testing, reflecting a significant unmet need for child-specific treatments [2]. Historically, pharmaceutical research has prioritised adults, leaving the dosing, safety, and acceptability requirements of children insufficiently addressed [99,100,105]. As a result, off-label prescribing, manipulation of adult medicines, and manual compounding remain common in paediatric care.

Children form a highly heterogeneous group with rapidly changing physiological characteristics that drive age-dependent variation in pharmacokinetics and pharmacodynamics, making single formulations unsuitable across all paediatric subpopulations [2]. Effective therapy further relies on adherence, which is heavily influenced by organoleptic attributes such as taste, smell, texture, and appearance [3]. Neonates often require liquid formulations despite challenges in stability, safety, and palatability, whereas older children may benefit from age-appropriate solid dosage forms that offer improved accuracy and acceptability [6]. Although compounding may answer the need for individual therapy, it is a laborious and time-consuming process, variable in quality, and often associated with risks of poor taste, instability, and inaccurate dosing, contributing to medication errors and hospital admissions [99]. Recognising these limitations, regulatory agencies have strengthened efforts to support paediatric formulation development and encourage innovation [2]. Paediatric medicines must comply with strict standards from the FDA, EMA, and WHO covering age-appropriate dosage forms, excipient safety, bioavailability, and overall product quality. The emergence of 3D printing introduces new regulatory considerations, including quality control, batch validation, and GMP compliance in decentralised environments such as hospital pharmacies [6].

In this section, an overview of currently available paediatric dosage forms is provided, highlighting categories where new therapeutic possibilities arise from 3D printing.

### 4.1. Liquid Oral Formulations

Oral liquid formulations—including solutions, emulsions and suspensions—are the most widely used dosage forms in paediatrics. They are easy to swallow and allow flexible dosing, making them suitable for neonates and infants. However, dose accuracy is a major concern; caregivers frequently struggle with measuring devices, and factors such as different spoon sizes or shaky hands can lead to under- or overdosing [105].

Solutions offer uniform dosing and do not require shaking. Still, they are limited to APIs with adequate solubility and may require co-solvents or surfactants that are unsuitable for young children [106]. Suspensions, used for poorly soluble drugs, must be shaken vigorously to ensure uniformity. They are frequently supplied as dry powders for reconstitution, which improves stability. However, once reconstituted, they have a limited shelf life and an increased risk of microbial contamination [107]. Syrups and elixirs improve palatability through their high sugar content or solvent systems; however, syrups may contribute to dental caries, and elixirs containing ethanol are not suitable for young children [108,109]. Oral drops allow the administration of small volumes but carry a higher risk of dosing errors due to their concentrated nature and variability in dropper performance [6].

Despite their widespread use, liquid formulations face several challenges, including poor palatability, chemical and microbiological instability, the use of unsuitable excipients, and frequent dosing inaccuracies. These limitations have driven interest in transitioning towards age-appropriate solid dosage forms—including 3D ones—which can provide improved dosing precision, stability, and personalised treatment options.

### 4.2. Solid Oral Dosage Forms

Despite advances in alternative drug delivery routes, the oral route remains the most preferred due to its convenience, safety, and accessibility [100,110]. Oral solid dosage forms remain the most widely used medicines worldwide. They are available in a broad range of shapes, sizes, and formats, making them suitable for many patient groups [111]. Their popularity stems from several advantages: they provide accurate, fixed dosing without the need for measurement, are easy to administer for patients able to swallow solids, offer good stability during storage and transport, and are cost-effective to manufacture. According to the European Pharmacopoeia, solid oral forms include powders, granules (including pellets), capsules, tablets, medicated chewing gums, and oromucosal preparations such as films and lozenges [112]. These formats encompass both swallowed and orally retained products, forming the foundation of oral pharmacotherapy. However, traditional “one-size-fits-all” dosing is often unsuitable for populations with variable physiology—such as children—where issues like swallowing difficulty, poor palatability, dose inflexibility, drug degradation in the gastrointestinal tract, and challenges related to tablet splitting can compromise therapeutic outcomes [110]. This is particularly problematic for medicines with a narrow therapeutic index.

Three-dimensional printing has introduced new possibilities for the creation of personalised oral solid dosage forms. It enables the fabrication of tablets, capsules, pellets, polypills, gastroretentive systems, and oral films with customised doses, tailored release profiles, and patient-friendly designs [100]. In the scientific literature, oral solid forms developed via 3D printing are commonly grouped into tablets, mini-tablets, pellets, capsules, and films, with specialised categories—such as chewables or gastro-retentive systems—discussed separately [112]

Together, conventional oral solid dosage forms and emerging 3D-printed variants offer a diverse platform for paediatric therapy, particularly as personalised medicine increasingly becomes attainable.

#### 4.2.1. Tablets

Tablets are the most widely used solid oral dosage forms, valued for their fixed dosing, stability, ease of administration, and cost-effective manufacturing [111]. However, their suitability in paediatrics is limited by swallowing difficulties, poor palatability, inflexible doses, and the risks associated with tablet splitting—particularly for drugs with a narrow therapeutic index [112].

Three-dimensional printing expands the role of tablets in paediatric therapy by enabling precise dose individualisation, multi-API combinations, and tailored release profiles, including immediate, delayed, or sustained release [2,10,100]. Complex internal architectures—such as lattice structures or porous matrices—can be engineered to control dissolution, enhance solubility of poorly soluble drugs, and improve bioavailability [3,10]. Child-friendly shapes, colours, and sizes can also be printed to support acceptability and adherence [3]. Importantly, 3D-printed tablets have demonstrated dose accuracy and release performance comparable to, or better than, those of conventional compounded liquid formulations [11].

For younger children, especially infants and toddlers, traditional tablets remain unsuitable. Mini-tablets (≤4–5 mm) offer a safer and more acceptable alternative: they are easier to swallow, well tolerated from as early as six months of age, and allow dose flexibility by adjusting the number of units administered [2,11,14,113]. Three-dimensional printing enables precise fabrication of mini-tablets with controlled size and release behaviour, including designs with internal channels to enhance dissolution [3,14]. Printed mini-tablets range from 1.5 to 10 mm in size. However, tiny tablets pose technical challenges due to printer resolution, cooling dynamics, and the risk of poor layer adhesion, making dose uniformity increasingly critical [6,11].

Three-dimensional printing enables the development of advanced multi-API tablet designs—such as dual-drug tablets, polypills, and multi-layer systems—that simplify complex regimens and improve adherence, particularly in paediatric patients requiring polypharmacy. By creating separate compartments or layers within a single dosage form, incompatible APIs can be physically isolated while still administered together, allowing precise, personalised dosing and flexible release patterns, including immediate, sustained, delayed, or pulsatile profiles [6,14,100]. Dual-drug tablets that incorporate two or more APIs within separate compartments to prevent incompatibilities, designed for simultaneous or sequential release. Additionally, multi-layer tablets incorporate two or more layers, each containing different APIs or designed with distinct release characteristics, combining rapid- and slow-release fractions. Polypills, on the other hand, are single-dose formulations containing multiple APIs in advanced forms, comprising up to five API compartments with independently controlled kinetics [3]. These 3D-printed systems have demonstrated the ability to reduce pill burden, enhance acceptability, and improve therapeutic outcomes in conditions such as HIV/AIDS, cancer, cardiovascular disease, diabetes, hypertension and chronic paediatric disorders where multiple medicines or time-dependent release patterns are required [100].

Overall, tablets—especially those produced via advanced 3D printing technologies—offer a highly adaptable platform for paediatric pharmacotherapy, enabling personalised dosing, tailored release profiles, and improved adherence that are not feasible with traditional manufacturing methods. When swallowing remains a barrier, 3DP also enables the production of chewable and fast-dissolving tablet formats, further improving acceptability [3,105].

#### 4.2.2. Pellets and Granules

Similarly to mini-tablets, granules and pellets are generally easy for children to swallow, making them widely used paediatric solid dosage forms [114]. Pellets are small, spherical, multiparticulate units—typically 0.5–2 mm in diameter—with uniform surface characteristics [115]. Granules, while generally larger, less spherical, and less uniform than pellets, share many of the same advantages, including ease of swallowing, flexible dosing when supplied as unit-dose packets, and good stability. The multiparticulate nature of pellets enables a more uniform distribution in the gastrointestinal tract, reducing the risk of local irritation or dose dumping, while allowing for dose flexibility by adjusting the number of units administered [115]. Due to their superior uniformity, flow, and coating properties, pellets are often preferred for the development of high-quality multiparticulate paediatric formulations. In paediatric applications, pellets are particularly attractive: their small size makes swallowing easier for children, especially when incorporated into sachets or sprinkled onto soft food, thus improving acceptability. Pellets can also be coated to provide effective taste masking or modified release, supporting both palatability and therapeutic control [115]. Regulatory and formulation reviews highlight pellets as a key dosage form for children, offering accurate dosing, adaptable release profiles, and high acceptability [116].

#### 4.2.3. Capsules

Capsules are well-established oral solid dosage forms consisting of a shell—traditionally gelatine or hydroxypropyl methylcellulose (HPMC)—that encloses powders, pellets, mini-tablets, or liquids. Their advantages include dose uniformity, ease of swallowing (particularly for older children), and the ability to combine multiparticulate systems within a single unit [117]. Capsules are also compatible with taste-masked pellets or mini-tablets, making them useful for paediatric patients who cannot tolerate large tablets. However, conventional capsules offer limited flexibility in customised release patterns and require manual filling or specialised equipment for dose individualisation [118].

Three-dimensional printing has introduced new possibilities for capsule design, enabling complex geometries, personalised dosing, and programmable drug release behaviours not achievable with traditional capsule shells. In 2015, Melocchi et al. produced the first fully 3D-printed capsule devices using hot-melt-extruded hydroxypropyl cellulose (HPC) filaments and fused deposition modelling (FDM). These capsules functioned as swellable, erodible, pulsatile-release systems, demonstrating controlled opening and time-dependent drug delivery—an approach particularly relevant for chronotherapy and diseases that benefit from delayed or burst-release patterns [119,120].

#### 4.2.4. Orodispersible Formulations

Orodispersible formulations—including orodispersible tablets (ODTs), orodispersible films (ODFs), lyophilisates, granules, and mini-tablets—play a growing role in paediatric therapy due to their ability to disintegrate rapidly in the mouth without requiring water, thereby improving ease of administration, adherence, and dosing accuracy [121,122]. The regulatory definitions for orodispersibility vary: the European Pharmacopoeia requires ODTs to disintegrate within 3 minutes, whereas the FDA specifies ≤ 30 seconds [112]. These dosage forms are beneficial for children, geriatric patients, and individuals with dysphagia. Across ODTs, ODFs, and sublingual systems, 3D printing offers patient-specific dosing, rapid disintegration, personalised shapes/colours, and improved acceptability, particularly for paediatric, geriatric, psychiatric, or dysphagic patients [2,100].

##### Orodispersible Tablets (ODTs)

ODTs are uncoated oral solid dosage forms that disintegrate rapidly in the mouth before swallowing, offering an important administration route for paediatric, geriatric, psychiatric, and dysphagic patients. Traditionally, ODTs are produced by compression, moulding, or lyophilisation [112].

The first—and currently only—FDA-approved 3D-printed medicine, Spritam^®^ (levetiracetam), is an ODT manufactured via ZipDose^®^ powder bed printing [100,112]. This technology has stimulated extensive research into 3D-printed ODTs using other methods, including binder jetting, FDM, and semi-solid extrusion, many of which meet Ph. Eur. requirements for disintegration time.

##### Orodispersible Films (ODFs)

ODFs are oromucosal solid preparations that disperse quickly in the mouth. Their dosing can be fine-tuned by adjusting the API concentration, changing the film thickness, or cutting the films to the desired size [6]. ODFs have become an appealing paediatric drug delivery system because they combine the benefits of liquids and solids while ensuring rapid dissolution in the oral cavity. The European ODFs are oromucosal solid preparations that disperse quickly in the mouth. Their dosing can be fine-tuned by adjusting the API concentration, changing the film thickness, or cutting the films to the desired size [6]. ODFs can be produced using inkjet (2D) or extrusion-based printing. Inkjet systems are ideal for low-dose APIs, whereas extrusion methods allow higher drug loads. Three-dimensional printing also enables advanced ODF designs. Bi-layer films incorporating mucoadhesive polymers (e.g., chitosan) have been developed to enhance adhesion and improve drug delivery [6]. Comparative studies have shown that 3D-printed ODFs exhibit faster disintegration and improved dose accuracy compared to 2D-printed films and oral powders, owing to their porous structure and larger surface area [11].

##### Sublingual Formulations

Sublingual formulations, which deliver medication via absorption under the tongue, offer a rapid onset and high bioavailability—particularly valuable for children or patients with swallowing difficulties. They are commonly used for APIs such as nitroglycerin, buprenorphine, zolpidem, ergotamine, and asenapine. Three-dimensional printing enables the precise, personalised production of sublingual tablets containing exact doses for each patient, supported by suitable excipients such as lactose, sucrose, dextrose, and mannitol [101]. Mucoadhesive polymers can be incorporated to prolong contact time and enhance absorption, making sublingual 3DP formulations a promising option for cardiovascular and psychiatric conditions.

#### 4.2.5. Chewable Formulations

Chewable dosage forms—including chewable tablets, gummies, gums, and lozenges—are increasingly important in paediatric, geriatric, and dysphagic populations because they eliminate the need to swallow tablets whole, offer pleasant taste profiles, and avoid many stability challenges associated with liquids [100,112,123]. Their relevance originates from the high prevalence of swallowing difficulties: approximately 1 in 11 primary care patients regularly struggle to swallow tablets or capsules, yet physicians often overlook this issue. This can lead to non-adherence or unsafe manipulation of the medications [123]. Chewable forms overcome these risks by allowing the dosage form to be broken down in the mouth before swallowing, improving comfort, safety, and acceptability.

Conventional chewables are typically produced by compression or moulding, but these methods can require multiple complex unit operations [124]. In contrast, 3D printing—particularly semi-solid extrusion (SSE) and food-grade printers—enables personalised chewable systems with soft, gummy-like textures suitable for thermolabile APIs [11]. Although 3D-printed chewables are often larger than standard tablets (8–84 mm), this is less problematic since they are intended to be chewed, not swallowed. Still, the FDA recommends a maximum dimension of 22 mm for chewable medicines [11]. Children reported to have a preference for 3D-printed chewables over ODTs or swallowable tablets [124]. Medical gummies encourage children to take their medication through their appealing appearance and organoleptic properties [14]. Extrusion-based 3D printing can produce highly individualised chewables, such as colourful, custom-shaped systems by embedding API-containing pastes within a gelatine matrix [2,3]. This modularity supports flexible dosing, age-appropriate strengths, and even the incorporation of multiple medicines into a single chewable unit [3]. Formulation, however, requires careful attention: unpalatable APIs demand strong taste masking; excessive hardness may damage teeth; and high levels of sweeteners or flavours (e.g., mannitol, sorbitol, dextrose, fruit flavours) are often required. Gelling agents, such as gelatine, cellulose derivatives, and starch, provide structure to the chewables, while pH modulators, such as citric acid, support stability and taste [100].

##### Chewable Tablets

Chewable tablets are oral dosage forms designed to be chewed before swallowing, producing a palatable residue that facilitates administration. According to the United States Pharmacopoeia, some chewable forms may be chewed optionally, but if the term “chewable” appears in the product name, the tablet must be chewed or crushed to ensure safe intake and proper API release. Chewable formulations provide a safer and more acceptable alternative to swallowable solids. In a clinical preference study, 79% of children aged 4–11 chose chewable tablets over other forms, highlighting their potential to enhance adherence significantly [2]. Traditionally prepared by moulding or extrusion, chewable tablets are particularly valuable in paediatrics, where individualised dosing is required; therefore, 3D printing has become an eagerly explored manufacturing approach for these formulations.

##### Gels

Gels, including hydrogels, are semisolid systems formed by polymeric networks such as cellulose derivatives, alginates, gums, carrageenans, or gelatine, and also offer flexibility in dose, shape, and mouthfeel, making them promising paediatric dosage forms [92]. They are easy to swallow and can be adapted into chewing gum-like medicines. While hydrogels are widely used in wound healing and biomedical engineering, interest in oral paediatric hydrogels produced by 3D printing is growing [92].

Furthermore, gummies—soft, chewable gel-based formulations—are increasingly popular in paediatrics because they are easy to chew, highly palatable, and visually appealing. They are elastic gels composed of gelling agents and water, most commonly gelatine; however, plant-based alternatives such as pectin and carrageenan are also increasingly used [92]. Sweeteners, flavours, emulsifiers, and oils improve taste, texture, and acceptance, while starch or coconut oil prevents sticking. Gummies are already widely used and commonly accepted as dietary supplements that contain vitamins and minerals. Recent studies have explored 3D-printed gummies, including the Lego-shaped gels containing paracetamol and ibuprofen [125]. These “drugmies”, i.e., drug-loaded gummies, resemble sweets, supporting adherence and allowing customisation of taste, shape, and API dose, benefits that make 3DP superior to conventional moulding for personalised paediatric therapy.

##### Medicated Chewing Gums

Medicated chewing gums are single-dose solid preparations composed of a gum base, intended to be chewed but not swallowed, to provide slow, sustained drug release through oral and transmucosal delivery [92]. Drugs released during chewing may be absorbed buccally or swallowed with saliva, enabling both local and systemic action. The chewing time (10–30 minutes) and chewing intensity significantly influence release kinetics. The formulation of chewing gums relies on elastomers, which provide the product with its mechanical properties and the desired texture for chewing. For paediatric patients, medicated chewing gums are beneficial due to their ease of administration without water, effective taste masking, improved acceptability for children and individuals with dysphagia, discreet dosing, and additional benefits such as stress reduction and oral care. Despite these advances and the extensive research conducted on medicated chewing gums, the number of studies on 3D-printed chewing gum is limited, with only a few early prototypes available [92].

### 4.3. Rectal Formulations

Paediatric rectal formulations—including suppositories, enemas, and rectal gels—remain vital alternatives when the oral route is impractical due to vomiting, dysphagia, seizures, or poor palatability. Rectal delivery is commonly used for both local and systemic drug delivery. It can facilitate the rapid absorption of certain active APIs, thereby partially avoiding first-pass hepatic metabolism, and is often feasible in low-resource or emergency settings [84,112]. Suppositories are the most widely used rectal dosage form in children and can be prepared with either lipophilic bases (e.g., hard fat) or hydrophilic bases (e.g., polyethylene glycol) that influence both melting behaviour and drug release kinetics [126]. Properly formulated suppositories exhibit acceptable tolerability and predictable pharmacokinetics. Paediatric suppositories commonly deliver antipyretics, antiemetics, anticonvulsants or laxatives [84,127]. However, rectal formulations must consider age-dependent anatomical differences, body temperature, and variability in colonic fluid volumes, which can influence dissolution and absorption [128]. Cultural acceptance also varies; however, caregivers often perceive rectal formulations as practical in cases of acute illness and when their children are unable to swallow medications [84]. Emerging research explores 3D printing of suppositories using semi-solid extrusion to customise dose, shape, melting behaviour, and multi-drug combinations, offering a pathway toward personalised paediatric suppositories [129].

### 4.4. Topical Formulations

Cutaneous and transdermal dosage forms play a crucial role in delivering drugs both locally to the skin and systemically. The European Pharmacopoeia defines liquid, semi-solid, and powder preparations for cutaneous application, as well as patches and medicated plasters. It requires sterility for products applied to large wounds or severely damaged skin [112].

Three-dimensional printing offers new possibilities not only for manufacturing customised drug-loaded patches and films, but also for wound dressings. The latter ones are not part of the topical formulations; however, they provide a shield for the damaged skin against infections, supporting the dermatological treatments. A recent literature survey identified 34 studies on 3D-printed cutaneous systems, ranging from non-gel and gel-based patches to advanced wound dressings; however, many publications did not clearly distinguish whether the systems delivered cutaneously or transdermally [112]. In the following, this review article discusses only the pharmaceutical forms containing active ingredients.

#### 4.4.1. Dermal Formulations

Paediatric dermal formulations—including creams, ointments, gels, lotions, pastes, patches, and medicated plasters—play a key role in treating local skin conditions. They are particularly valuable in infants and young children, who often present with dermatological conditions such as eczema, diaper dermatitis, and bacterial or fungal infections, and who may have difficulty swallowing systemic therapies [130]. Compared with adults, children have a higher skin surface area–to–body weight ratio and a thinner stratum corneum, which can increase dermal absorption and the risk of systemic exposure; therefore, paediatric topical formulations require careful selection of API concentration, excipients, and dosing frequency [131]. Alcohols, fragrances, strong preservatives, and penetration enhancers may cause irritation or sensitisation in young children, making excipient choice especially critical [132]. Semi-solid preparations are the most commonly used in paediatrics, providing ease of application, occlusion when needed, and the ability to soothe or protect the skin. In acute or severe conditions, such as severe diaper rash, burns, or atopic flares, gels or medicated dressings provide targeted delivery while minimising systemic exposure [130].

#### 4.4.2. Transdermal Patches

Transdermal patches are non-invasive drug formulations that are easy to administer and can be used by anyone, anywhere. However, only APIs with specific physicochemical characteristics—typically a low molecular weight (<400 Da), adequate lipophilicity, and a low effective dose (<20 mg/day)—can be formulated successfully as transdermal systems, and they must be non-irritant and non-sensitising to the skin [133]. Although these dosage forms are convenient, the unique biopharmaceutical properties of young children must be considered: due to their thinner stratum corneum and higher surface-area-to-body-weight ratio, paediatric patients are particularly vulnerable to unintentional overdosing or systemic toxicity [134]. Recent advances in three-dimensional (3D) printing have expanded the potential of transdermal systems in paediatrics by enabling personalised patches with adjustable size, drug load, and release profiles. Three-dimensionally printed transdermal patches—including reservoir-based systems, matrix patches, and microneedle-assisted patches—can be fabricated with precise geometries to tailor dose to body weight or developmental stage, optimising local treatment while reducing systemic exposure [101,112]. Extrusion- and inkjet-based printing methods enable controlled deposition of drug-polymer matrices, producing patches with custom thickness, surface area, and drug distribution, while ensuring reproducible adhesion and flexibility. For paediatric use, 3D printing also enables the incorporation of child-friendly shapes and colours to improve acceptability without altering pharmacokinetics [100].

More advanced paediatric applications include 3D-printed microneedle patches, which provide minimally invasive transdermal delivery and can enhance the delivery of drugs that are otherwise unsuitable for passive diffusion across the skin. These microneedles can be printed to dissolve or swell upon application, providing a safe and pain-free administration for children while enabling controlled or sustained release of APIs [100]. Three-dimensionally printed microneedle patches are a promising but not yet pharmacopoeia–listed dosage form [112].

### 4.5. Pulmonary Formulations

Pulmonary drug delivery is a cornerstone of paediatric therapy, particularly for asthma, cystic fibrosis, bronchopulmonary dysplasia, and acute respiratory infections. Nebulisers, pressurised metered-dose inhalers (pMDIs), dry powder inhalers (DPIs), and soft mist inhalers (SMIs) each offer distinct advantages and limitations for children, whose respiratory physiology and inhalation abilities differ significantly from those of adults [135]. Age-appropriate selection is crucial to ensure the correct device type is chosen. Infants depend on nebulisers and face-mask delivery; toddlers gradually transition to spacer-assisted pMDIs; school-aged children can learn correct inhaler technique; and adolescents generally achieve reliable DPI performance. Flavouring, aerosol temperature, mask fit, device noise, and treatment time strongly influence acceptability and adherence in paediatric populations [136].

Emerging research in 3D printing explores personalised paediatric inhalation therapy, such as custom-shaped spacers, optimised airflow geometries, and patient-specific adaptors, to improve lung deposition and usability. Prototype 3D-printed inhaler components and add-on devices demonstrate the potential for low-cost, customised pulmonary delivery solutions customised to children’s anatomy and breathing profiles [137]. However, not only inhaler devices, but also inhaled particles, can be 3D printed. In a recent collaboration, Wostry et al. used high-resolution multi-photon 3D printing to fabricate carrier particles with precisely defined geometries for dry powder inhalers [138].

### 4.6. Parenteral Formulations

Parenteral dosage forms are sterile preparations for injection, infusion, or implantation that bypass external barriers to deliver drugs directly into the body. These preparations encompass sterile solutions, powders, suspensions, emulsions, injectable gels, implants, intravitreal products, and drug-eluting stents. Sterility is a core requirement for all these formulations [112].

Injectable and infusion-based products are essential in paediatric therapy when rapid, reliable, or continuous drug delivery is required. They bypass swallowing difficulties and enable precise weight-based titration, but remain invasive, often painful, and must be administered by trained healthcare professionals—an important consideration in neonates and young children, who have heightened pain sensitivity [139]. Parenteral administration is required in acute scenarios, such as unconsciousness, dehydration, resuscitation, renal replacement therapy, acute analgesia, sepsis, or immunisation, as well as in chronic disease management that requires biologics. Careful formulation, appropriate administration techniques and pain-reduction strategies are critical to ensuring safe, effective, and child-friendly parenteral therapy [140].

Paediatric injectables include intravenous (IV), intramuscular (IM), subcutaneous (SC), and occasionally intraosseous routes, selected based on age, vascular access, drug characteristics, and urgency [141]. Formulations must consider excipient safety, osmolality, pH, injection pain, volume limits, and developmental differences in muscle and adipose tissue [113].

Although liquid injectables and infusions cannot be manufactured directly via 3D printing, 3D-printed systems are designed for parenteral administration and may offer future advantages in paediatric therapy. Three-dimensional printing is most relevant for solid parenteral dosage forms, despite the need for semi-solid states during fabrication. Traditionally, solid implants for sustained local or systemic release are produced using hot-melt extrusion (HME) to form rod-shaped implants or moulding techniques for specialised geometries, while highly customised devices, such as vascular stents, rely on unconventional methods, such as laser cutting and subsequent coating [112]. Experimental research has further explored injectable 3D-printed micro-scaffolds and shear-thinning hydrogels that can be delivered through a needle [142,143]. Beyond drug products, 3D printing is increasingly used to prototype paediatric injection-support tools such as neonatal micro-dosing adapters, syringe connectors, and infusion-line components [144]. While none of these innovations are yet approved for routine paediatric use, they highlight the growing potential of 3D printing to support safer, more precise, and personalised parenteral drug delivery in the future.

### 4.7. Special Drug Delivery Systems

#### 4.7.1. Nipple Shield Delivery System (NSDS)

Nipple shield delivery system (NSDS) is an innovative paediatric formulation designed to deliver medicines or nutrients to breastfeeding infants. The mother inserts a small dose form—typically a mini-tablet—into a compartment within the silicone shield; thus, during suckling, the infant receives the active ingredient dissolved in the milk stream. This approach allows medication delivery without disrupting natural feeding routines and is particularly useful for neonates who cannot swallow other dosage forms [145].

#### 4.7.2. Reservoir-Based Devices

Reservoir-based devices are another group of child-friendly formulations that enhance palatability and simplify administration. These include reservoir feeding bottles, pacifiers, pulp spoons, and medicated straws.

In reservoir feeding bottles and pacifiers, the API is placed in a chamber connected to a hollow nipple, delivering medication as the infant suckles. These systems are suitable even for newborns [146].

Pulp spoons contain a single premeasured dose of dry API; upon mixing with water, a paste forms that can be administered directly on the spoon [146].

Medicated straws are filled with taste-masked particles (granules, pellets, or crystals). Children receive the dose by drinking a predetermined volume of liquid through the straw. While convenient and non-invasive, drug release can be influenced by the temperature and speed of fluid intake, which may affect dose accuracy [147].

#### 4.7.3. Solid Dosage Pen (SDP)

A solid dosage pen (SDP) contains a drug-loaded rod that is incrementally advanced by a screw mechanism. The rod is cut into individualised segments at the point of administration, enabling precise dose personalisation. Formulations may include standard compressed-rod systems or effervescent formulations that disperse rapidly upon ingestion. However, challenges remain, including the need for taste masking of bitter APIs, specialised packaging, and the technical difficulty of formulating stable drug-loaded rods [148].

Together, these specialised paediatric delivery systems aim to improve adherence, minimise distress, and support accurate dosing in infants and children—particularly those unable or unwilling to swallow traditional oral dosage forms.

Potential combination of these systems with 3DP may lead to personalised designs, rapid prototyping, custom ergonomic shapes, integrated drug compartments, and precise control over dose and release characteristics. In populations where swallowing difficulties, anxiety, or sensory sensitivities limit the use of traditional tablets, 3D printed adaptations of these devices provide an optional path toward safe, acceptable, and individualised paediatric therapy.

Table 4 summarises the main administration characteristics of the paediatric drug formulations [149,150,151].

## 5. Three-Dimensional Printing Technologies

Three-dimensional printing, also known as additive manufacturing, involves the layer-by-layer construction of three-dimensional objects from a computer-aided design (CAD) model. Its principal advantage lies in the ability to produce complex geometries that would be difficult—or even impossible—to achieve using conventional manufacturing methods [152]. Over the past decades, 3D printing has transformed nearly every industrial sector, including medicine, dentistry, prosthetics, food, art, aerospace, and construction [153].

The evolution of three-dimensional (3D) printing began in the 1980s with a pioneering work on photopolymerisation and laser-based prototyping, ultimately leading to the development of stereolithography (SLA) by Charles Hull in 1987 and the invention of selective laser sintering (SLS) and fused deposition modelling (FDM) shortly thereafter [14,154,155,156]. Although initially restricted to industrial prototyping due to high costs, the field expanded rapidly with the introduction of low-cost printers through the RepRap initiative in 2005 [157]. Since the 2010s, advances have enabled printing with diverse materials—including polymers, metals, ceramics, and biomaterials—supporting applications ranging from engineered tissues to customised drug delivery systems [14,158]. In the pharmaceutical field, 3D printing has emerged as an additive, layer-by-layer manufacturing approach capable of producing dosage forms with complex geometries, tunable release profiles, and highly individualised attributes [10,14].

Three-dimensional printing has become a promising tool for addressing long-standing limitations of conventional paediatric formulations by enabling personalised, on-demand dosage forms with precise control overdose, geometry, and release profile. Initial developments often focus on APIs lacking suitable oral formulations, which are available only as poorly tolerated liquids or large tablets, suggesting that the current formulations could be improved [11]. Across multiple studies, 3D-printed printlets and other oral forms have demonstrated accurate, flexible dosing, excellent content uniformity and stability, acceptable mechanical properties and dissolution, and compliance with pharmacopeial standards, favoured by both children and healthcare professionals compared with conventional tablets [11,124]. The technology allows quick, bedside adjustment of dose, drug combinations, release profiles and sensory characteristics such as size, shape, colour, flavour, and texture. It also allows the fabrication of diverse dosage forms—including mini-tablets, ODTs, ODFs, chewables, polypills, suppositories, gastroretentive systems, and other non-oral dosage forms—customised to the patients’ age, clinical needs, and individual preferences [2,3,6,10,84,99,100,110,112]. In paediatrics, this individualisation is particularly valuable for low or adjustable doses, combination therapies, improved swallowability and palatability, and enhanced adherence [2,10]. Quality and feasibility studies confirm that 3D-printed dosage forms can meet pharmacopeial standards and deliver optimised drug release and safety; however, regulatory frameworks, GMP standards, and pharmacopoeial methods specific to 3D-printed medicines are still in development. Furthermore, the widespread adoption of 3D printing in paediatric pharmacy faces regulatory, technical, and logistical hurdles [6,14,124].

A wide range of 3D printing technologies is now used in the fabrication of medicinal products, including inkjet-, nozzle-, electromagnetic-, and ultrasonic-based systems, each with specific advantages and technical constraints [11,14]. Technique selection depends heavily on API characteristics, excipient compatibility, target dose, and desired drug-release behaviour. At the same time, it is worth noting that not all methods are universally suitable for paediatric formulations [11]. Using computer-aided design and manufacturing (CAD/CAM), digital blueprints are converted into machine-readable instructions that guide the precise layer-by-layer deposition of materials, enabling the fabrication of dosage forms with controlled internal structures, separate compartments, and adjustable shapes and sizes [110,112]. This design flexibility reduces reliance on traditional processing steps such as milling, granulation, and compression, making 3D printing particularly advantageous for small-batch or point-of-care production, although challenges remain in particle-size control, nozzle performance, and the preparation of starting materials [11].

**Figure 4:**
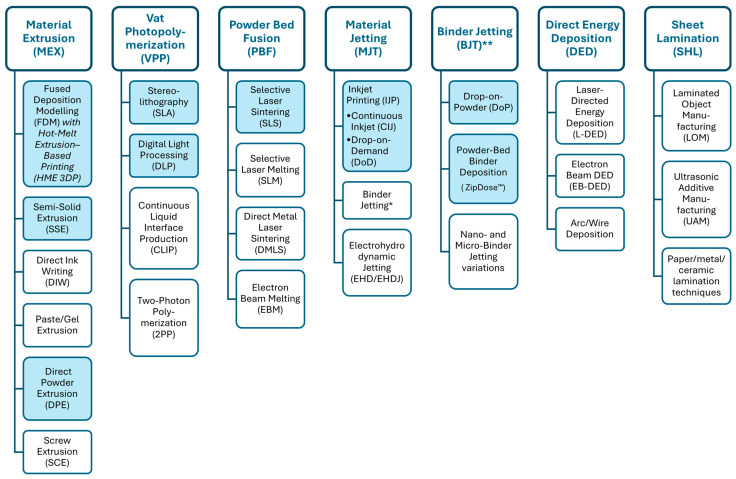
Three-dimensional printing technologies grouped according to the ASTM categories. Highlighted boxes indicate the pharmaceutically most relevant 3D printing methods. * In industrial terminology, Binder Jetting is distinct from Material Jetting, although in pharmaceuticals “DoP inkjet” is sometimes described under MJT. ** Pharmaceutical literature often groups Drop-on-Powder/Spritam^®^ ZipDose under inkjet-like techniques, but formally, ASTM classifies Binder Jetting as a separate category.

According to the American Society for Testing and Materials (ASTM), 3D printing technologies are classified into seven categories: Material Extrusion (MEX), Vat Photopolymerisation (VPP), Powder Bed Fusion (PBF), Material Jetting (MJT), Binder Jetting (BJT), Directed Energy Deposition (DED), and Sheet Lamination (SHL) (see Figure 4 and Table 5) [2,10,99]. In pharmaceutical research, the most commonly investigated techniques include Fused Deposition Modelling (FDM), Semi-Solid Extrusion (SSE), Direct Powder Extrusion (DPE), Selective Laser Sintering (SLS), and Stereolithography (SLA) [10,14]. Additional technologies, such as Digital Light Processing (DLP), Inkjet Printing (IP), Direct Ink Writing (DIW), UV-curable Inkjet Printing, Continuous Liquid Interface Production (CLIP), Melt-Electrowriting (MEW), Deep Eutectic Solvents (DES), and Electrohydrodynamic Jet (E-Jet) printing, are actively being developed to broaden formulation options [100,110]. The following listing describes the most suitable 3D printing technologies for pharmaceutical applications.

### 5.1. Material Extrusion (MEX)

Extrusion-based 3D printing is the most widely applied technology in pharmaceutical formulation development, particularly for paediatric drug delivery systems. Common techniques include fused deposition modelling (FDM), semi-solid extrusion (SSE), and direct powder extrusion (DPE), all of which produce dosage forms by extruding molten or semi-solid materials through a nozzle, using heated or unheated systems, depending on the formulation requirements [11]. Material extrusion (MEX) technologies are considered highly suitable for small-batch and personalised medicine manufacturing due to their flexibility, accessibility, and the availability of pharmaceutical-grade excipients [99,101].

A critical element in filament-based extrusion is the feeding and processing of polymers, excipients, and APIs through twin-screw extruders, where parameters such as feeding rate, specific feed load, and residence time distribution strongly influence material flow and uniformity. Accurate dosing systems, such as vibrating trays or gravimetric feeders, are essential to ensure consistent mixing and homogeneity [6]. The optimisation of pharmaceutical filaments requires extensive offline and inline characterisation, including analytical assessments of physical properties, drug distribution, release behaviour, and process stability, to ensure that final 3D-printed products meet the required quality attributes for clinical use [6].

#### 5.1.1. Fused Deposition Modelling (FDM)

##### Synonym Names: Filament Extrusion (FE), Fused Filament Fabrication (FFF)

**Figure 5:**
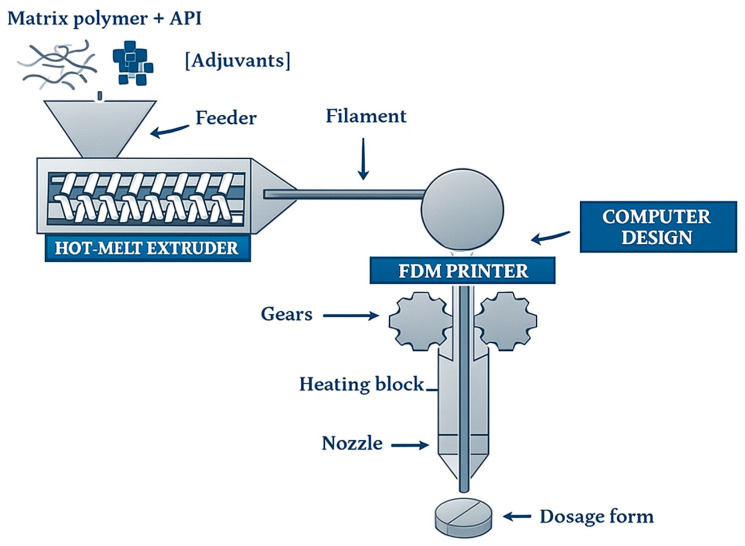
The schematic figure of the Fused Deposition Modelling combined with Hot-Melt Extrusion-Based Printing technique.

Fused deposition modelling (FDM) (see Figure 5) is a material-extrusion 3D-printing technique in which a drug-loaded filament—usually produced by hot-melt extrusion (HME)—is reheated and softened in the printer nozzle, then mechanically pushed forward to deposit material layer by layer [112]. Initially patented by Scott Crump in 1989, FDM quickly became a key technology for personalised medical devices and customised drug delivery systems [14]. Its popularity arises from its low cost, ease of use, and ability to produce complex oral dosage forms with customised release profiles [11,100].

In pharmaceutical applications—particularly in paediatrics—FDM enables precise spatial arrangement of drug-polymer matrices, allowing for controlled-, sustained-, or immediate-release dosage forms, as well as innovative child-friendly geometries [3,10,14]. Combining FDM with HME offers additional advantages, such as improved solubility through amorphous solid dispersions and the ability to create multi-API combinations. However, its dependence on high temperatures (typically 120–250 °C) limits its applicability for thermostable APIs and thermoplastic polymers, such as polyvinyl alcohol, polylactic acid, polyvinylpyrrolidone, and hydroxypropyl cellulose, as well as Eudragit derivatives [92,111]. Low-temperature FDM can expand this range, but thermal sensitivity remains a significant limitation.

A critical challenge for paediatric medicines printed via FDM is ensuring uniform filament diameter and homogeneous API distribution, as any variability directly affects dose accuracy and may violate pharmacopeial requirements—especially problematic in low-dose paediatric formulations [6]. Additional hurdles include nozzle clogging, variable feed rates, degradation of thermolabile drugs, and print-to-print variability [6,10]. Despite these challenges, FDM remains one of the most researched and promising pharmaceutical 3D-printing techniques due to its flexibility, reproducibility, and suitability for personalised paediatric dosage forms [6,101].

##### Hot-Melt Extrusion–Based Printing (HME)

Filament extrusion via hot-melt extrusion (HME) (see Figure 5) is a critical upstream step in producing drug-loaded filaments for Fused Deposition Modelling (FDM). In line with the ASTM additive manufacturing classification, FDM is a material extrusion–based 3D printing technique. In contrast, HME is not an additive manufacturing technique, but rather a continuous material-processing method used to prepare drug-loaded extrudates, most commonly filaments. In pharmaceutical practice, however, HME is frequently integrated into the FDM workflow because the extruded filaments produced by HME serve as the feedstock for FDM printing. The HME itself does not perform layer-by-layer deposition and therefore falls outside the ASTM 3D-printing categories.

In the HME process, polymer–API blends are carefully prepared and processed under controlled conditions to ensure uniformity in drug distribution, filament diameter, and mechanical properties [6]. HME can be used in pharmaceutics to create solid dispersions, thereby improving the solubility of poorly water-soluble drugs and providing effective taste masking [3]. It also serves as a precursor to 3D-printing steps [11].

Technically, the success of HME relies heavily on efficient polymer melting and homogeneity. Polymers with favourable melt viscosity and thermal conductivity, along with optimised screw design and kneading elements, ensure smooth flow and uniform mixing. To maintain consistent filament diameter—critical for paediatric dose accuracy—melt pumps are often incorporated into the printer to stabilise pressure and throughput, preventing fluctuations that could compromise content uniformity [6].

#### 5.1.2. Semi-Solid Extrusion (SSE, EXT)

Synonym names: Syringe Extrusion (SE), Pressure-Assisted Microsyringe (PAM) Printing, Pneumatic Extrusion, Direct Ink Writing (DIW), Paste Extrusion/Gel Extrusion.

**Figure 6:**
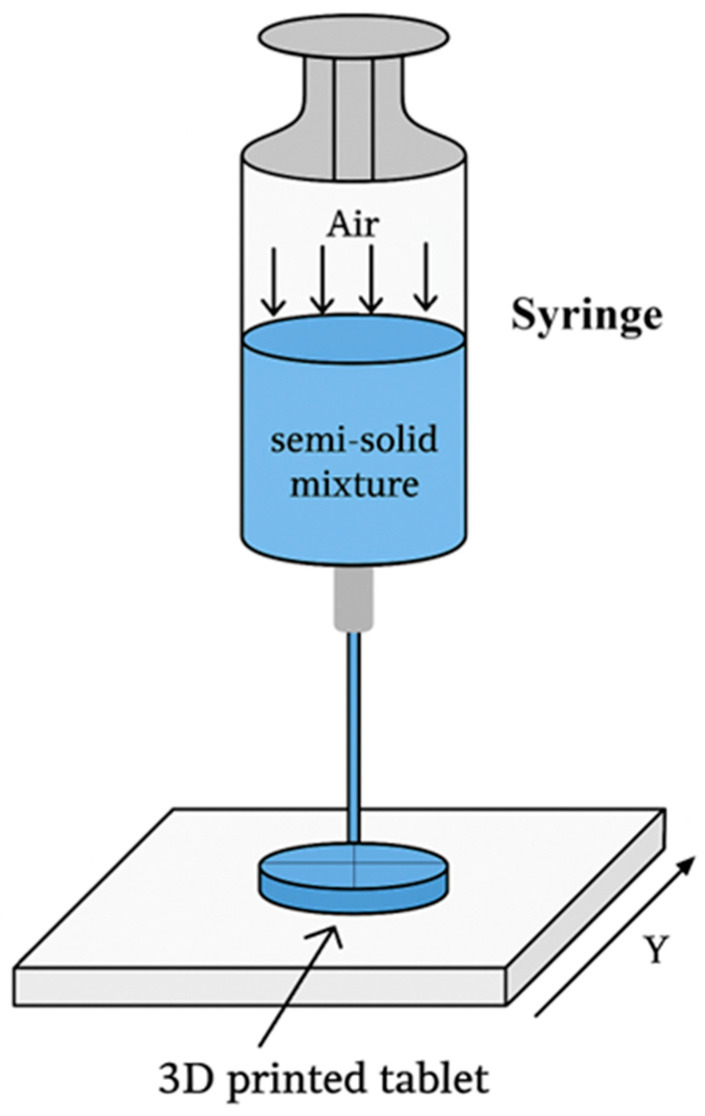
The schematic figure of the Semi-Solid Extrusion technique.

Semi-Solid Extrusion (SEE) (see Figure 6)—also known as Syringe Extrusion (SE), Pressure-Assisted Microsyringe (PAM) Printing, Direct Ink Writing (DIW), Pneumatic Extrusion, or Paste/Gel Extrusion—is a material-extrusion technique in which a semi-solid formulation (gel, paste, or slurry) is extruded from a syringe-like cartridge using pneumatic or mechanical pressure [112]. Because materials are processed in a semi-solid state and typically at low or no heat, SSE is particularly attractive for pharmaceuticals, as it accommodates a broad range of APIs and excipients, including temperature-sensitive compounds, and is well-suited to clinical and point-of-care settings [10,11]. After deposition, SSE-printed structures solidify by cooling, solvent evaporation, or post-printing drying, which may introduce additional processing steps and sometimes cause shrinkage or deformation [100,112].

SSE enables the fabrication of diverse paediatric-friendly dosage forms such as chewables, gummies, immediate- and controlled-release tablets, gastroresistant systems, and especially orodispersible films, which often show improved dose accuracy and faster disintegration than 2D-printed films [11,14,100]. Its ability to extrude viscous gels supports the development of hydrogels and lipid-based systems for controlled release and permits the creation of personalised shapes—including child-friendly or cartoon-like designs—to enhance acceptability [14]. SSE has also been explored for innovative concepts such as cereal-based printed formulations containing ibuprofen and paracetamol to improve adherence in paediatric hospital settings [14].

However, SSE presents limitations: prints typically have low resolution, the method requires stringent control of viscosity and rheology, and many formulations need lengthy drying times, making the technique less suitable for emergency hospital use [2,10]. When organic solvents, such as acetone or dimethyl sulfoxide, are used to enable printability, residual-solvent limits must be carefully controlled in particular cases for paediatric patients [2]. SSE printed semi-solid structures may collapse if they are not sufficiently hardened; high-viscosity materials require larger nozzles, which further reduces precision [3,100]. Despite these challenges, SSE remains a versatile, mild-processing 3D-printing platform capable of producing personalised, age-appropriate formulations—particularly designed for paediatric use [100].

#### 5.1.3. Direct Powder Extrusion (DPE)

**Figure 7:**
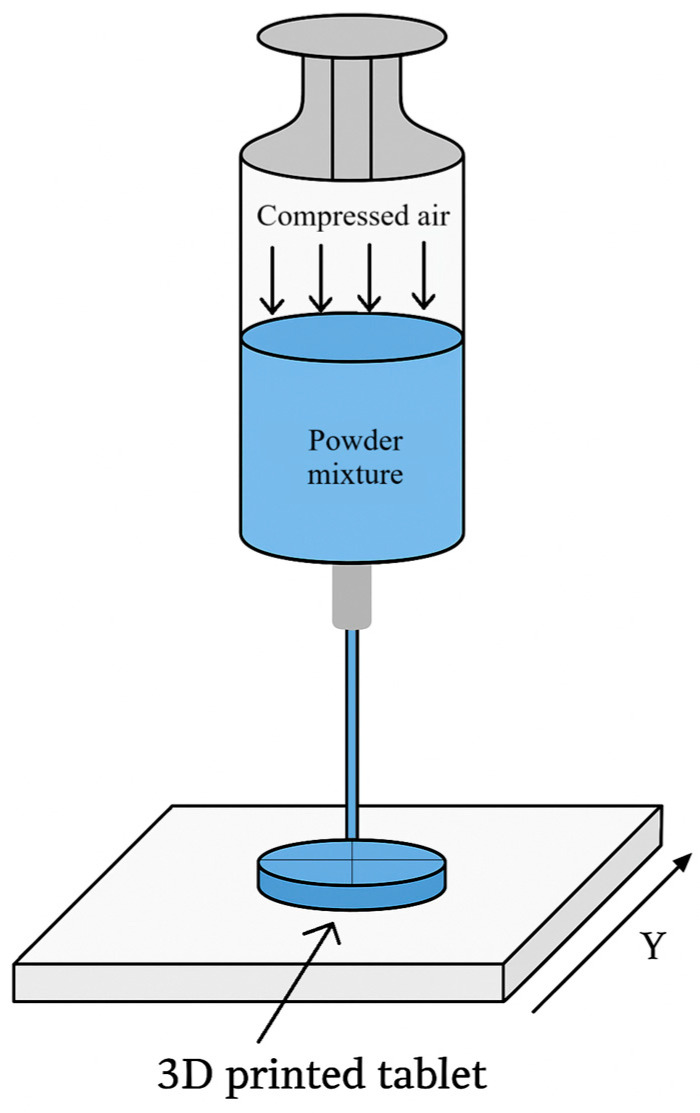
The schematic figure of the Direct Powder Extrusion technique.

Direct Powder Extrusion (DPE) (see Figure 7) is an emerging 3D-printing technique that processes powder blends of APIs and excipients directly, eliminating the need for solvent-based processing or pre-manufactured drug-loaded filaments [100]. In DPE, powders are fed into a hopper and conveyed into a heated single-screw extruder located in the printhead, where they are fused and extruded through a nozzle to form the final solid dosage form [14]. This single-step approach overcomes several limitations of filament-based FDM, enabling higher API loading, reducing thermal exposure by avoiding two separate heating steps, lowering development costs, and broadening the range of printable formulations—particularly those unsuitable for filament production [2].

DPE supports the manufacture of both immediate- and sustained-release systems, can generate solid amorphous dispersions to enhance the solubility and bioavailability of poorly soluble drugs, and allows the design of complex geometries and multilayer drug-delivery systems [2,100]. The absence of solvents simplifies processing, eliminates drying steps, and reduces stability concerns associated with residual solvents. As DPE requires only small quantities of powder, it shows strong potential for clinical and hospital-pharmacy use, where personalised tablets can be produced rapidly and cost-effectively [110]. However, the technique is limited by its unsuitability for heat-sensitive APIs, challenges in selecting compatible excipient–API blends, and the potential for less uniform API distribution compared to hot-melt extrusion [14,100,110]. Overall, DPE represents one of the most innovative and promising one-step 3D-printing strategies for personalised solid oral dosage forms in pharmaceuticals [100].

#### 5.1.4. Screw Extrusion (SCE)

Screw extrusion (SCE) refers to 3D printing processes in which solid materials—typically powders or granules—are conveyed and forced through a nozzle by a screw-driven mechanism, rather than relying solely on pneumatic or mechanical pressure. This category includes many dry powder extrusion (DPE) systems, where the screw ensures continuous feeding, mixing, and compaction of the formulation during printing. SCE setups may use either single-screw or twin-screw configurations, each influencing material flow, shear, and print quality. SCE accommodates a wide range of formulations and enables precise control over print parameters, making it increasingly relevant for pharmaceutical applications [112].

### 5.2. Vat Photopolymerization (VPP)

Vat Photopolymerization (VPP) is a liquid-solidification 3D printing method in which a light source—typically a laser or projected light—induces local photopolymerisation within a vat of liquid resin, solidifying selected regions to build the object layer by layer [11,112]. VPP encompasses technologies such as stereolithography (SLA/SL), digital light processing (DLP), and two-photon polymerisation, all of which enable the precise fabrication of pharmaceutical dosage forms and medical devices. In a pharmaceutical context, SLA and DLP are the most widely used approaches, offering high accuracy and fine structural resolution [99]. Emerging volumetric VPP methods, such as tomographic and multi-beam printing, promise increased printing speed and improved mechanical properties [99].

#### 5.2.1. Stereolithography (SLA)

**Figure 8:**
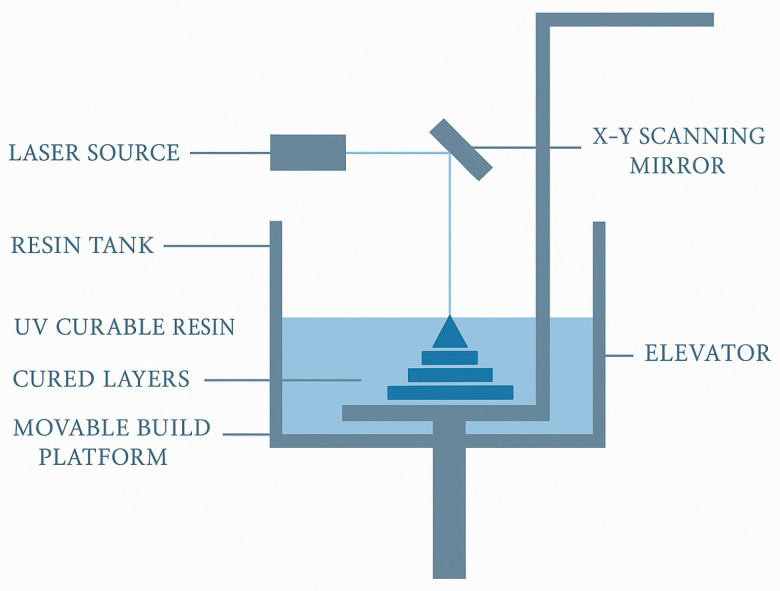
Schematic figure of the Stereolithography technique.

Stereolithography (SLA) (see Figure 8) is a light-based, solvent-free 3D printing technique in which a UV laser selectively solidifies liquid photopolymer resin layer by layer. Developed in the early 1980s by Carl Deckard and Joe Beaman, SLA offers high accuracy, rapid printing, and excellent surface quality, making it suitable for complex drug delivery systems and sustained-release formulations [10,14,100].

SLA relies on photopolymerisable, UV-curable resins, which are often limited in number and not always available as pharmaceutical-grade materials, raising concerns regarding toxicity, biocompatibility, and regulatory acceptability [14,92,110]. Its dependence on light exposure makes the method unsuitable for photosensitive or thermosensitive drugs; however, it avoids the thermal degradation risks associated with high-temperature techniques [14,92].

Despite material constraints, SLA excels in producing high-resolution, geometrically intricate structures, supporting applications such as implants, microfluidic devices, multi-drug tablets, and soft devices for controlled release [3,100].

Overall, SLA offers precision and speed; however, its broader pharmaceutical use is limited by resin toxicity, restricted polymer choices, and stability issues, suggesting that different 3DP techniques may be better suited for specific drugs and clinical applications [10,110].

The newest form of SLA 3DP is volumetric stereolithography. Using this method, complex, high-quality 3D objects can be printed within seconds [159].

#### 5.2.2. Digital Light Processing (DLP)

**Figure 9:**
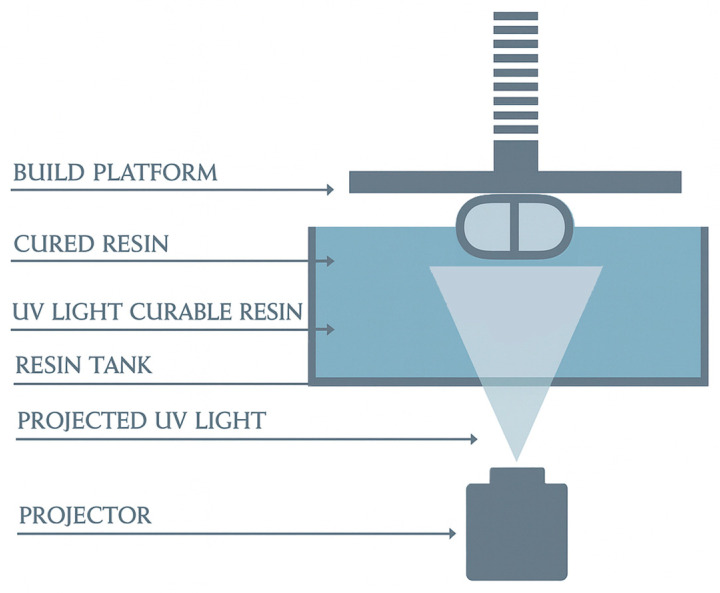
Schematic figure of the Digital Light Processing technique.

Digital Light Processing (DLP) (see Figure 9) is a vat-photopolymerisation technique based on light-induced photopolymerisation of photosensitive resins, operating similarly to stereolithography (SLA) but distinguished by its use of a digital micromirror device [75]. The digital micromirror device contains thousands of movable micro-mirrors that rapidly toggle between on/off positions, enabling simultaneous, whole-layer exposure. This feature makes DLP faster and more cost-efficient than SLA while maintaining high print quality. DLP requires low-viscosity, photo-crosslinkable materials; however, fabricating soft or highly flexible dosage forms can be challenging due to deformation under the weight of subsequent layers [75]. The technique allows precise control over parameters such as wavelength, exposure time, and UV intensity; however, UV exposure may lead to API photodegradation, posing risks to stability and safety [75].

In pharmaceutical applications, DLP enables rapid curing of photopolymer resins to produce high-resolution drug-delivery devices, including detailed microneedle arrays for transdermal administration [100]. Its capacity for fast, layer-wide curing supports high-throughput customisation and iterative formulation development [100].

#### 5.2.3. Continuous Liquid Interface Production (CLIP)

In 2015, the continuous liquid interface production (CLIP) process was introduced, enabling faster print speeds than previous approaches. It allows the production of objects in minutes rather than hours [160].

### 5.3. Powder Bed Fusion (PBF)

Powder Bed Fusion (PBF) uses a powder bed similar to binder jetting, but instead of applying a liquid binder, it fuses powder particles through thermal energy, typically via a laser or electron beam, to build objects layer by layer [100,113]. Common PBF processes include Selective Laser Sintering (SLS) and Selective Laser Melting (SLM), where heat is applied in a highly controlled, localised manner to create solid structures without the need for binding fluids [112].

#### Selective Laser Sintering (SLS)

**Figure 10:**
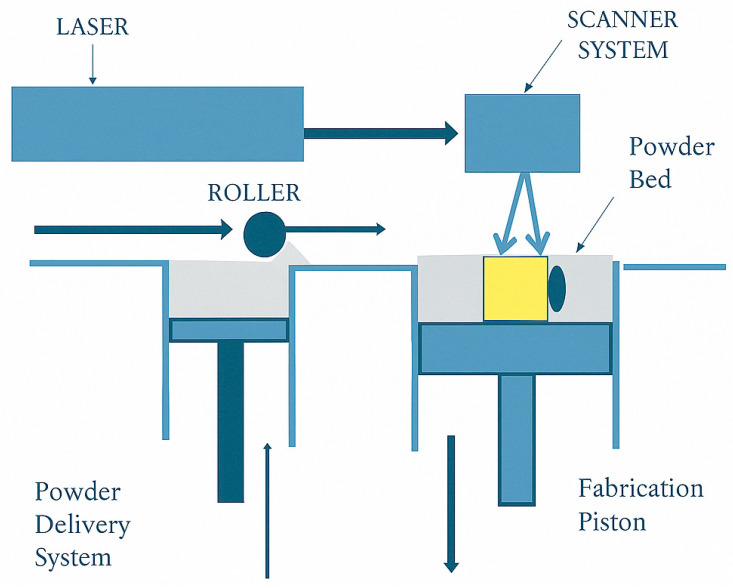
The schematic figure of the Selective Laser Sintering technique.

Powder-solidification 3D printing, which includes drop-on-powder (DoP) and selective laser sintering (SLS), creates solid dosage forms by either binding powder particles with a liquid or fusing them through heat [11]. SLS (see Figure 10) uses a laser to sinter powder particles layer by layer selectively: the laser traces the pattern of each layer on the powder bed, a roller distributes fresh powder, and the cycle repeats until the object is fully formed [10,14]. The process begins by heating the build chamber to just below the melting temperature of the powdered material. Once this temperature is reached, a roller deposits and evenly spreads the first powder layer. A laser is then guided by a scanning system to follow the programmed geometry, locally heating the powder above its melting point so that particles fuse into a solid layer [161]. After each layer is completed, the laser pauses, a new layer of powder is spread, and sintering continues, building the object from the bottom up. When printing is complete, the entire build chamber must cool before the part can be removed, which is often a time-consuming final step [161]. This process yields high-resolution, highly porous structures, making SLS promising for customised drug delivery systems [100], though it requires high energy, powerful laser components, and post-processing, and is unsuitable for photo- or thermosensitive APIs [10,14].

Because SLS relies on thermal fusion, commonly used powders include thermoplastic polymers such as polyamides or polystyrenes [3]. However, these industrial-grade materials limit pharmaceutical applications due to biocompatibility concerns [110]. Nonetheless, SLS provides a solvent-free manufacturing route that makes control over product porosity and geometry [92].

The SLS method is used for more durable drug delivery systems, including implants and controlled-release dosage forms. This additive manufacturing process is also well-suited for fabricating implants with complex geometries, such as scaffolds for tissue restoration [162].

### 5.4. Material Jetting (MJT)

Material jetting (MJT) encompasses 3D printing processes in which objects are formed by selectively depositing tiny droplets of liquid material—such as molten substances, photopolymer resins, or solvent-based inks—that solidify either spontaneously or through curing methods, including drying or UV exposure [99,113]. In pharmaceuticals, MJT includes inkjet printing, drop-on-demand (DoD), and electrohydrodynamic (EHD) printing, all of which allow highly precise spatial placement of liquid droplets.

Inkjet printing (IJP) is the most widely explored MJT method. It functions as a Drop-on-Demand (DoD) system, depositing picolitre-sized droplets (1–70 pL; 10–50 μm) onto a substrate with high positional accuracy [11,92]. Due to this precision, it is especially suitable for low-dose APIs, personalised dosing, and multi-drug deposition, though the total printable drug load remains limited [11]. Inkjet printing can create complex, multilayered pharmaceutical structures, print APIs onto films, stents, microneedles, or even nails, and is being explored for integrated drug-delivery/monitoring systems using printed biosensors [100].

In a study, inkjet printing was used to fabricate orodispersible films (ODFs), and both 2D inkjet-printed and 3D-extruded films demonstrated better dose precision than compounded oral powders; 3D-printed films disintegrated faster due to their higher surface area [11].

### 5.5. Binder Jetting (BJT)

Powder-solidification 3D-printing techniques form solid dosage forms by binding or fusing powder particles, most commonly through binder jetting (BJT)—also known as drop-on-powder (DoP) or powder-bed inkjet printing—and selective laser sintering (SLS) [11]. In contrast to material jetting, where droplets themselves form the object, BJT deposits a liquid binder onto a powder bed, selectively agglomerating particles to build up successive layers [99,112]. The binder may be present in the powder, the liquid phase, or both, and further post-processing—such as drying, oven curing, or infiltration—may be required to strengthen the typically fragile printed structures [100].

BJT/DoP is particularly suitable for high-dose formulations and for producing highly porous orodispersible dosage forms [11,110]. The best-known example is Spritam^®^, the first FDA-approved 3D-printed medicine, produced using Aprecia’s DoP-based ZipDose^®^ technology, which enables the rapid disintegration of levetiracetam tablets up to 1000 mg [11,100]. Research groups have further adapted DoP systems to print colourful, child-friendly ODTs with quality attributes comparable to Spritam^®^ [11]. However, DoP printers require solvents, and the resulting tablets often have low mechanical strength, limiting their use in sustained-release applications [11,111].

In pharmaceutical BJT, print resolution depends on droplet size, powder granularity, and binder penetration; typical powder-bed requirements include particle size < 1 μm, viscosity < 20 cP, and surface tension ~50 mN/m [3]. Piezoelectric print heads, used in continuous or drop-on-demand modes, offer better droplet control than thermal heads and support a wider range of ink formulations [3]. BJT has been used to create controlled-release, fast-dissolving, and delayed-release tablets; however, complex structures may require support material, and print accuracy is influenced by powder spreading, agglomeration behaviour, and binder diffusion [10,100]. Overall, powder-solidification techniques—especially BJT/DoP—enable high-dose, rapidly disintegrating, and highly porous paediatric dosage forms, but face limitations in mechanical strength, solvent handling, and suitability for extended-release formulations.

## 6. Three-Dimensionally Printed Paediatrics-Specific Product and Process Requirements

Developing paediatric-appropriate formulations requires defining a range of key attributes within the paediatric quality target product profile (pQTPP), including the route of administration, age range, target release profile, dosage form, dose and dose flexibility, patient acceptability, handling and preparation steps, administration device, excipient safety, child-resistant packaging, stability, manufacturability, and patient access. Three-dimensional printing offers unique advantages for several of these attributes—notably dose accuracy and dose flexibility, customised release profiles, adaptable dosage forms, improved patient acceptability, and personalised administration strategies [11]. Three-dimensional printing supports child-centred design by enabling customised shapes, colours, and personalised doses, thereby enhancing patient compliance through acceptance and adherence [14].

For pharmaceutical applications, 3D printers must meet exceptionally high standards for precision, reproducibility, and temperature control, as well as the ability to process pharmaceutical-grade excipients without additional modification, handle multi-material printing, provide sufficient throughput, and comply with GMP [14]. Ensuring quality in paediatric 3D-printed medicines requires tight control of critical quality attributes (CQAs), including accurate layer deposition to maintain dose uniformity, appropriate mechanical strength, controlled surface smoothness affecting dissolution, and carefully regulated porosity and density to achieve predictable release profiles [2]. In practice, defects such as gaps, deformation, or cracking may still occur due to suboptimal processing conditions, emphasising the need for high-quality print heads, stable material flow, and technology-specific adaptations. Paediatric medicines demand exceptional safety, stability, and cleanliness; developing suitable printers requires collaboration between manufacturers, formulation scientists, excipient suppliers, and regulators. At the same time, printers must remain user-friendly, supported by intelligent software that can recommend printing parameters, predict dosage form behaviour, and enable remote adjustment. As pharmaceutical 3D printing is in its early stages, measuring material behaviour and achieving consistent print quality remains challenging, necessitating continuous innovation in materials, equipment, and validated manufacturing protocols. Additionally, specialised training for operators, clinicians, and pharmacy personnel is essential to ensure the safe and reliable production of personalised paediatric dosage forms [10].

The development of 3D-printed paediatric medicines requires a structured understanding of the factors that determine product quality, safety, and therapeutic performance. Building on the principles of paediatric formulation science and quality-by-design, this chapter outlines the Critical Quality and Performance Attributes (CQPAs) that guide the selection of an appropriate dosage form and printing technology for children. These attributes encompass dose accuracy and flexibility, drug release behaviour, palatability and acceptability, product size and material choice, as well as safety, stability, cost-effectiveness, production time, scalability, and reproducibility. Together, these considerations frame the design space for personalised paediatric dosage forms and ensure that 3D-printed medicines can meet clinical needs while supporting reliable, child-friendly therapy.

### 6.1. Dose Accuracy

Ensuring dose accuracy is one of the most critical challenges in 3D-printed paediatric formulations. Studies assessing oral 3D-printed paediatric doses typically evaluated accuracy and precision through content uniformity testing or drug content assays, with a few relying on mass-variation methods [11]. While assays can demonstrate production precision, they do not always confirm that the intended target dose is achieved [163]. Variability can arise from fluctuations in extrusion rate, nozzle temperature, and layer deposition inherent to 3D printing, leading to uneven API distribution within or between batches. To address these risks, emerging approaches propose real-time, non-destructive inline monitoring tools—such as NIR or Raman spectroscopy—to track API dispersion and process stability during printing [6]. This need for continuous process control is heightened by the small batch sizes typical of decentralised hospital or pharmacy-based 3D printing, where traditional destructive quality testing is impractical [6].

### 6.2. Dose Flexibility

Dose flexibility is one of the greatest benefits of 3D printing for paediatric medicines. Unlike traditional manufacturing, where tablets come in fixed strengths that often require splitting, dilution, or extemporaneous mixing, 3D printing enables precise, on-demand dose adjustment to suit a child’s age, weight, clinical condition, or pharmacokinetic needs. Usually, dose flexibility is achieved by scaling the dimensions or internal volume of the digital design, which reliably correlates with API content across both low- and high-dose formulations. Other methods include altering drug concentration within the printable matrix or—particularly in inkjet and drop-on-solid techniques—modifying ink concentration, print resolution, print area, or the number of deposited layers. However, studies indicate that relying solely on the number of drug-containing layers is less accurate because printers can only deposit whole layers, leading to rounding errors when fractional layers would be required. Overall, 3D printing offers unprecedented, finely tuned dose personalisation for paediatric patients, with the potential to eliminate safety risks associated with tablet splitting or liquid dilution [11].

### 6.3. Drug Release Profile

Three-dimensional printing offers control over drug release profiles, enabling the design of immediate-, delayed-, sustained-, or multipart release systems within a single dosage form—an essential advantage for paediatric therapy, where age-dependent pharmacokinetics and swallowing ability often necessitate tailored release behaviours. Precise dosage and time-release profiles can be achieved by modifying factors such as shape and size, and combining the APIs with excipients [111]. Studies show that increasing the overall dimensions of a printed tablet slows drug release, as larger structures extend diffusion pathways and reduce surface-area-to-volume ratios [11]. Infill percentage, which represents the proportion of solid polymer to internal air, generally has only a minor influence on dissolution but can still be used to fine-tune release kinetics when combined with other design parameters [164].

Beyond geometry, material selection and formulation adjustments provide another layer of release control: matrix composition, polymer grade, binder type, hydrophilicity, and excipient ratios can be modified to achieve predictable immediate or sustained release. Techniques such as FDM, SLA, and inkjet printing further allow the creation of multi-compartment designs, layered structures, or barrier coatings that modulate drug diffusion [165]. For paediatric applications, this flexibility enables the development of child-specific release profiles—such as slower release for neonates with immature metabolism or rapid release for acute conditions—while also supporting the creation of taste-masked, age-appropriate oral forms [106].

### 6.4. Palatability and Acceptability

Patient acceptability—driven mainly by palatability—is a significant determinant of adherence in paediatric therapy. Children’s willingness to take a medication depends on sensory factors such as appearance, colour, shape, smell, taste, aftertaste, and mouthfeel [11,124]. Three-dimensional printing enables highly appealing dosage forms, including flavoured chewables, gummies, jellies, chocolate-based systems, and units shaped like cartoons or LEGO^®^ bricks. These designs can significantly improve acceptance but also raise concerns about confusion with candy and accidental ingestion. Studies consistently show that children prefer colourful, familiar shapes, smooth surfaces, and chewable textures. Traditional circular tablets remain the most accepted, with unconventional geometries sometimes reducing swallowability [11].

Three-dimensionally printed formulations can also enhance acceptability through improved quality and precision, as studies have shown that these formulations produce smoother surfaces, better dose uniformity, and more visually appealing tablets than subdivided or compounded alternatives. Younger paediatric groups prefer smaller units and mini-tablets. Diameters as small as 2 mm are accepted even by neonates [11].

Taste masking is crucial, as children are sensitive to bitterness and rely on both taste and smell. Three-dimensional printing supports multiple taste-masking strategies: incorporating sweeteners and flavours, forming inclusion complexes (e.g., cyclodextrins, polyelectrolytes), using high-viscosity polymers (HPMC, guar gum, sodium alginate) to slow dissolution in saliva, or applying polymer matrices and coatings that delay release until after swallowing [6]. Personalised flavour systems—such as FlavoRiTe, which allows children to select their own flavours—can further enhance acceptability [10]. Amorphous or molecularly dispersed APIs can enhance palatability but may increase bioavailability, necessitating careful dose control to mitigate the risk of overdose [11].

Chocolate-based paediatric formulations are emerging as an appealing strategy to improve acceptability and mask the bitterness of many APIs, thus encouraging medicine intake in children [2]. Chocolate is particularly suitable due to its pleasant taste, smooth texture, and emotional familiarity. Its bioactive components, such as cocoa polyphenols, have been associated with antioxidant and cognitive benefits [3]. Karavasili et al. demonstrated that a chocolate–corn syrup mixture can be optimised to produce stable, self-standing printed structures containing paracetamol or ibuprofen, with a 1:1 chocolate-to-syrup ratio offering the best balance between printability, handling, and drug loading. This approach enabled rapid fabrication of multilayered shapes—from stars to cartoon figures—in just minutes, demonstrating strong potential for paediatric therapies requiring improved adherence [3,92]. However, formulation design must consider potential food–drug interactions arising from the chocolate’s caffeine and tyramine content, which may interfere with drugs such as MAO inhibitors, theophylline, or ciprofloxacin [3].

Regulatory agencies are now encouraged to develop guidelines for swallowability, taste masking, acceptable geometries, and bioavailability for 3D-printed paediatric medicines [6]. These should define safe tablet sizes, textures, disintegration times, and patient-centric design requirements, including palatability testing and in vitro/in vivo evaluation of release and absorption profiles. While challenges remain—particularly with overly rough or unconventional shapes—emerging 3D-printed chewables, orodispersible systems, and sublingual forms offer strong potential to improve paediatric adherence when designed with safety and acceptability in mind.

### 6.5. Size

The size of paediatric dosage forms is a critical determinant of swallowability and, consequently, adherence. Orodispersible and chewable formulations—often produced via 3D printing—represent promising alternatives to conventional tablets for school-aged children and adolescents, but their suitability depends heavily on maintaining appropriately small dimensions [11]. Even when a dosage form is designed to disintegrate rapidly or be chewed, excessively large units can still pose swallowing challenges [124]. Healthcare professionals have therefore raised concerns about maximum acceptable sizes, and the FDA continues to apply uniform size limits (the largest dimension of a tablet or capsule intended to be swallowed whole should not exceed 22 mm) across all oral solid dosage forms [166]. In this context, 3D printing offers a distinct advantage: it enables precise control over geometry, allowing tablets to be miniaturised, customised to individual age groups, and engineered for improved handling. As such, the ability of 3D printing to fine-tune size without compromising dose or functionality makes it especially valuable for paediatric formulation design.

### 6.6. Material Selection

Material selection is a central determinant of quality and safety in 3D-printed paediatric formulations. The choice of polymers, plasticisers, fillers, disintegrants, surfactants, release modifiers, solvents, colourants, and flavourings must align with the intended drug release profile, the selected printing technology, and the physicochemical characteristics of the API [100]. Polymers such as hydroxypropyl cellulose (HPC), hydroxypropyl methylcellulose (HPMC), ethyl cellulose (EC), hydroxypropyl methylcellulose acetate succinate (HPMCAS), polyvinylpyrrolidone (PVP), and methacrylate derivatives are widely used due to their printability and ability to stabilise APIs, including through solid dispersions that improve the solubility of poorly water-soluble drugs. However, high polymer loads, exceeding 50% *w*/*w*, require careful evaluation in paediatric populations, whose immature metabolic and gastrointestinal systems may respond differently to prolonged polymer exposure [14].

Excipients may cause allergic reactions, posing a significant challenge in finding the best ones [150]. The Safety and Toxicity of Excipients for Paediatrics (STEP) database is a free resource that compiles the safety and toxicity information of excipients for paediatric medicine [167,168]. Therefore, excipients must be closely investigated for paediatric safety. Solvents used in orodispersible formulations, residual monomers in photopolymer resins, and thermally degraded by-products from FDM technology can pose toxicity risks. Several common excipients accepted in adults—such as propylene glycol, benzyl alcohol, polysorbates, PEGs, and even widely used polymers like PVP—lack sufficient paediatric safety data or are known to cause toxicity in neonates. Processing constraints further complicate material choice: thermolabile APIs may degrade during hot-melt extrusion, and many photopolymerizable materials used in SLA or DLP printing are not on the FDA’s Generally Recognised as Safe list of substances considered safe for consumption, limiting their suitability for medicines [92].

Stability and solid-state transformations, especially in amorphous solid dispersions produced by FDM, depend on polymer–API miscibility and manufacturing conditions. A wide range of printable material forms—such as powders, filaments, resins, pellets, or granules—exists, but innovation in truly paediatric-appropriate materials remains limited. Challenges include ensuring biodegradability, drug compatibility, mechanical integrity, and the absence of toxic residuals across printing methods [14].

Regulatory considerations form an additional layer of complexity. Paediatric drug development must comply with the EMA and c E11(R1) guideline on the clinical investigation of medicinal products in the paediatric population, which requires age-appropriate dosing, excipient safety assessments, and paediatric-specific pharmacokinetic–pharmacodynamic studies [6].

### 6.7. Safety

Excipients pose a critical safety challenge in paediatric formulations. Infants and young children are highly susceptible to hypersensitivity reactions and metabolic toxicities from substances such as parabens, propylene glycol, benzyl alcohol, polysorbates, PEGs, and even lactose [92]. Because 3D printing enables precise formulation control, allergenic or unsafe excipients can be easily excluded and replaced, thereby improving safety. Nevertheless, the small internal components of 3D printers complicate cleaning and sterilisation, and inappropriate cleaning methods (e.g., prolonged ultrasonic treatment) may damage equipment or leave residues that affect product quality [10].

Robust evaluation of safety and efficacy is essential before clinical adoption. Regulatory agencies emphasise patient acceptability, accurate dosing, and reliable therapeutic performance, particularly in paediatrics, where swallowability and adherence are major determinants of treatment success. Professional training for pharmacists, clinicians, and machine operators is required to ensure safe handling and standardised production. Hospital and pharmacy-based 3D printing units will also need adapted GMP frameworks, real-time monitoring, and rigorous quality systems to manage small-batch, personalised production [10].

Operator safety must be achieved through enclosed print chambers, effective air filtration, and automated contamination-detection systems to minimise exposure to APIs and vapours [6]. Waste management presents additional challenges: although 3D printing can reduce manufacturing residues, defective batches cannot be recycled due to contamination risks and loss of material properties [92].

Every printed batch requires thorough quality control, including verification of dose accuracy, dissolution behaviour, mechanical integrity, and sterility. Facilities must maintain validated equipment, securely store digital design files, and maintain complete traceability records to support recalls and regulatory compliance. Collectively, these measures ensure that personalised 3D-printed paediatric medicines meet the same safety and efficacy standards as traditionally manufactured products [100,124].

### 6.8. Stability

Stability is a significant limitation of compounded oral liquids, which often have short shelf lives, unknown degradation pathways, unpleasant taste, the presence of potentially harmful solvents, and a high risk of dosing errors at home [11]. In contrast, 3D-printed solid dosage forms offer a promising stability advantage: because they contain no water and have restricted molecular mobility, they are less vulnerable to physical, chemical, and microbiological degradation. However, despite this theoretical benefit, long-term stability data for 3D-printed tablets are not available yet. Key aspects—including residual solvent levels, degradation behaviour, and robustness under paediatric handling conditions—are still insufficiently characterised. The stability of intermediate products (e.g., filaments, pastes, or printable powders) is also not well established, yet it is crucial for the practical and cost-effective operation of pharmacies. These intermediates must remain stable enough for storage before printing to ensure predictable quality and minimise waste. Comprehensive stability studies are urgently needed before routine paediatric use becomes feasible [11].

### 6.9. Cost-Effectiveness

Cost-effectiveness remains a significant uncertainty in the implementation of 3D printing for paediatric drug production. While the technology offers clear advantages, its economic feasibility remains uncertain [11,124]. Healthcare professionals believe that automation and precise on-site manufacturing can lower long-term costs by eliminating overproduction and improving efficiency; however, they also express concerns about the substantial initial investment required for high-grade printers, maintenance, specialised training, and consumables [14,124]. Personnel costs, raw materials, packaging, and disposable components continue to add to operational expenses. Moreover, the personalised nature of 3D-printed medicines introduces regulatory challenges: customised formulations may require additional evaluation or approval, potentially increasing development time and cost [100]. Although 3D printing has the potential to enhance adherence and reduce waste, robust economic studies are still needed to determine whether its benefits outweigh the financial burdens in real-world paediatric healthcare settings.

### 6.10. Production Time

Production time is a key functional factor in evaluating the feasibility of 3D printing for paediatric medicines. One of the major advantages is the ability to produce personalised dosage forms rapidly and on demand at the point of dispensing, thereby significantly shortening lead times for children requiring customised therapies. Studies demonstrate that 3D printing can be highly efficient for small-batch manufacturing—for example, twenty-eight tablets (one month of treatment) were produced in approximately eight minutes, illustrating the speed and resource efficiency achievable compared to conventional compounding [124]. However, despite these benefits, production times can still be comparatively long compared to high-throughput industrial manufacturing, limiting efficiency for large-scale use [112].

### 6.11. Scalability

Although the FDA approval of Spritam^®^ in 2015 demonstrated that 3D-printed medicines can be manufactured and commercialised at scale, broader industrial adoption remains challenging. Current 3D printing technologies are inherently slow due to their layer-by-layer fabrication process, making them less efficient than traditional high-throughput manufacturing methods for producing large-volume products. The strengths of 3D printing lie in creating small, personalised batches rather than supplying mass markets [2]. Scalability is further limited by the restricted range of printable, pharmaceutical-grade excipients, as materials must be biocompatible, stable, and GMP-compliant. However, many APIs and conventional excipients are incompatible with current printing platforms [100]. High equipment costs, limited material availability, and the need for specialised expertise also hinder widespread implementation, particularly in clinical settings [110]. To enable industrial expansion, advances in printing speed, cost-effective systems, expanded excipient libraries, and standardised regulatory frameworks are essential. Continuous innovation in modelling software and process control will also be required to balance customisation with reproducibility and regulatory compliance. The successful integration of 3D printing into large-scale pharmaceutical production depends on achieving a balance between customisation and standardisation, managing costs, and continuously improving the technological processes [2].

### 6.12. Reproducibility

Regulating 3D-printed pharmaceuticals requires a customised approach because each printing technology has distinct mechanical and chemical properties, even when producing the same formulation [92]. Unlike traditional large-batch manufacturing, where uniformity is ensured through sampling-based quality control (QC), 3D printing often produces small batches or even single personalised units, making conventional QC methods insufficient. Variability in printer calibration, material composition, and environmental conditions can significantly affect dose accuracy, mechanical strength, and dissolution behaviour. Consequently, regulatory frameworks must incorporate in-process monitoring and real-time release testing to verify the quality of every printed unit. Standardised protocols—such as spectroscopic analysis and automated verification systems—are essential to ensuring consistent drug content, stability, and performance across individually printed doses [6].

## 7. Industrial Implementation of 3D-Printed Medicines

### 7.1. Spritam^®^ Orodispersible Tablets

Research into pharmaceutical 3D printing expanded rapidly in the 2010s, and as a result, in 2015, the FDA approved Spritam^®^—the first and still only 3D-printed medicinal product on the market. Spritam^®^ is a levetiracetam-containing orodispersible tablet intended for patients aged 4 years and older with epileptic seizures developed by Aprecia Pharmaceuticals (Blue Ash, OH, USA). The product is manufactured using ZipDose^®^ technology, a high-porosity binder jetting (BJT) process. At BJT, a binding fluid is deposited onto loosely packed powder layers. The process creates a highly porous structure that allows the tablet to disperse in the mouth with minimal liquid in under 10–11 seconds, making administration particularly suitable for paediatric, elderly, and dysphagic patients [14,99,112,120].

Spritam^®^ tablets offer high drug-loading capabilities, with fixed strengths of 250 mg, 500 mg, 750 mg, and 1000 mg levetiracetam. Although not a personalised product, its approval demonstrated the commercial feasibility of producing fast-dissolving, high-dose ODTs using 3D printing at an industrial scale [99].

Following the approval, a research team developed multi-head DoP/BJT printers capable of producing colourful, cartoon-like levetiracetam ODTs. These experimental tablets met quality specifications for hardness, friability, dispersion, and drug release, performing comparably to Spritam^®^ [11].

Despite the rapid expansion of scientific literature since 2015, no additional 3D-printed medicinal products have yet been approved by the FDA or the European Commission (via EMA). Despite several companies having 3DP-based development projects in their pipelines, Spritam^®^ remains the leading marketed 3D-printed oral dosage form and continues to guide research into orodispersible systems, particularly regarding printing methods, excipient selection, and disintegration performance aligned with pharmacopoeial ODT standards [112].

### 7.2. Triastek’s 3DP Pipeline

Although Spritam^®^ remains the only FDA-approved 3D-printed medicine to date, several pharmaceutical companies are now advancing 3D-printed drug products toward clinical evaluation and eventual market entry. Among these, Triastek (Nanjing, China) is the most prominent, with multiple products progressing through the Investigational New Drug (IND) pathway [112].

Triastek has received FDA IND clearance for at least four 3D-printed drug candidates, enabling interstate shipment for clinical studies in the United States [99,113]. These products are manufactured using the company’s Melt Extrusion Deposition (MED^®^) platform, a specialised, highly controlled form of fused deposition modelling (FDM). MED^®^ allows precise control of the geometry and distribution of both APIs and excipients, supporting multi-compartment structures and sophisticated release kinetics that were not achievable with the ZipDose^®^ binder-jetting technology used for Spritam^®^. Furthermore, this technique allows large-scale production of personalised drug formulations [110]

One of Triastek’s most advanced candidates, T19, which contains tofacitinib, a JAK inhibitor, is designed for the treatment of rheumatoid arthritis and has already entered clinical trials. The formulation uses a timed-release structure to align with circadian patterns of pain and inflammation. For this candidate, a New Drug Application (NDA) submission is anticipated in the near future [3]. Additional candidates include:

- T20—Designed for cardiovascular diseases;
- T21—Engineered for targeted delivery to the colon to treat disease, including ulcerative colitis [110];
- T22—A fourth MED-based product intended for pulmonary arterial hypertension [99].

These developments demonstrate the growing feasibility of using 3D printing to create dosage forms with precisely tuned release profiles, which is particularly valuable for paediatric patients who require flexible dosing and improved tolerability.

Despite the rapid expansion of academic research—thousands of publications over the past decade—human clinical data remain scarce, and Spritam^®^ remains the only approved product 9 years after its launch. While this slow translation into marketed medicines may seem disappointing, it likely reflects the normal caution of industry, as companies rarely publish early trial data. The steady rise of 3D-printed medical devices, along with multiple IND approvals, suggests that pharmaceutical products are likely to follow suit [112].

Looking ahead, the demand for individualised medicine—including paediatric patients with unmet dosing needs—is expected to drive further adoption. The prospect of on-demand, in-hospital, or pharmacy-based production of personalised dosage forms is emerging, offering tailored strength, release profiles, and palatability. As regulatory frameworks evolve and more clinical evidence accumulates, it is anticipated that additional 3D-printed medicines will reach the market in the coming years.

### 7.3. Current Clinical Trials on 3DP Drug Formulations

The approval of Spritam^®^ demonstrated the feasibility of 3D printing pharmaceuticals and their acceptability even in paediatric medicines [3].

Among industrial pipelines, Triastek has developed several candidates using its proprietary melt extrusion deposition (MED) platform [99]. Its product, T19, received clearance for an Investigational New Drug (IND) application from the FDA and is progressing through clinical trials, with a New Drug Application (NDA) anticipated. MED allows precise tuning of drug release, which is advantageous for tailoring paediatric doses [3]. Merck is also exploring powder jetting and material extrusion technologies for producing oral solids for clinical trials, with the goal of achieving future GMP-compliant 3D printing (3DP) manufacturing [3].

The patent landscape includes high-dose levetiracetam 3DP designs, fused filament fabrication-based tablet production systems, multilayer Triastek dosage forms with programmable release, compartment-based controlled-release constructs, and 3DP orally disintegrating tablets containing amlodipine besylate or olanzapine via inkjet printing [92].

Most clinical evidence comes from academia. Early preclinical and clinical studies have shown promising results, including bioequivalence, reproducible drug distribution, and increased acceptability [99]. The first in-human trial evaluated 3DP chewable isoleucine printlets by FabRx at the University of Santiago de Compostela [93]. A subsequent study in six children assessed hospital-printed amino acid formulations, demonstrating therapeutic equivalence to compounded preparations and high patient acceptability [92,124].

Further studies on paediatric metabolic disorders, including maple syrup urine disease, confirmed that 3DP chewable tablets are feasible and acceptable, and for the first time, enabled the combination of multiple amino acids into a single printlet. This reduced the dose burden and enhanced quality of life. Three-dimensional printing was also evaluated as an alternative to manual levothyroxine subdivision for infants. These findings highlight the increasing potential of point-of-care 3D printing of medicines [99].

Despite progress, major challenges complicate clinical trial planning for 3DP medicines, including changing regulatory frameworks, ethical and legal issues, patient recruitment hurdles, administrative delays, costs, the unique complexities of paediatric trials, and a lack of experience [99].

### 7.4. Regulatory Landscape and GMP Challenges

Despite rapid advances in extrusion-based 3D-printing technologies, regulatory frameworks and Good Manufacturing Practice (GMP) standards for 3D printers, software, and pharmaceutical-grade input materials remain underdeveloped. Current pharmacopoeias lack analytical methods tailored explicitly to 3D-printed tablets, and existing standards are only applicable to conventional oral solid dosage forms [14]. Although many polymers used in 3D printing are recognised as safe in adults, their suitability for children must be independently confirmed, emphasising the need for paediatric tolerance studies and the development of new polymeric excipients [14].

At present, the FDA regulates 3D-printed products under existing frameworks, but specific GMP guidelines for 3D-printed pharmaceuticals have not yet been established; only medical devices have defined pathways since 2017 [14]. This gap is particularly problematic for decentralised, point-of-care manufacturing in hospitals and pharmacies, where challenges arise around batch consistency, operator training, sterility, raw material quality, and process validation. Traditional GMP systems—designed for large-scale batch production—are poorly suited to personalised, small-batch 3D-printed medicines [6].

Core regulatory questions remain unresolved, such as whether each printed unit should be treated as an individual batch or whether multiple identical doses produced within a short time frame can be grouped for validation. Clear criteria for batch definition, testing requirements, and stability assessment are essential, especially when paediatric doses are printed on demand in clinical settings [6]. To ensure safety and reproducibility, regulatory agencies must establish GMP frameworks specific to 3DP, covering material traceability, equipment calibration, cleaning validation, and process standardisation. Cleaning validation is one of the most critical GMP challenges. Because 3D printers may handle multiple APIs, residues in nozzles, print heads, heating chambers, and reservoirs can lead to cross-contamination. Techniques such as FDM and inkjet printing require validated cleaning protocols with predefined residual limits for GMP compliance [6]. Material traceability is equally important: filaments and inks must demonstrate consistent drug loading, diameter, and stability, supported by Certificates of Analysis, batch records, and robust in-process and post-print testing [6].

Consumer-grade 3D printers are unsuitable for pharmaceutical use due to their uncleanable geometries, exposed lubricated parts, and lack of enclosure or filtration. GMP-compliant printer design requires API-contact surfaces made from approved, easily cleanable materials without joints or undercuts, capable of withstanding repeated sanitisation [6]. Ensuring sterility and stability presents additional regulatory hurdles. No standardised sterilisation guidelines exist for 3D-printed medicines produced outside traditional cleanrooms. Regulatory agencies must therefore develop protocols to prevent microbial contamination in hospital-based production [6].

More broadly, regulatory agencies must expand and adapt existing frameworks to ensure the safe and effective deployment of 3DP in paediatric medicine—including decentralised manufacturing, paediatric-specific standards, and global harmonisation [6]. Based on that, different printing platforms vary in precision and reproducibility; therefore, printer-specific validation protocols are needed. The absence of 3DP-specific GMP pathways continues to slow commercialisation and limits clinical translation [110].

Several regulatory bodies are taking steps to modernise the 3DP frameworks. The FDA is exploring strategies to regulate point-of-care 3DP with a focus on quality assurance and risk-based oversight. The Medicines and Healthcare Products Regulatory Agency in the United Kingdom supports decentralised, modular manufacturing of personalised medicines through its new regulations. In the EU, the EMA published a 2025–2027 work plan for its Quality Innovation Group QIG that includes a "Questions and Answers" document covering 3DP and decentralised manufacturing to clarify regulatory expectations [99]. The potential of 3D printing to produce personalised paediatric formulations also intersects with US frameworks such as the Best Pharmaceuticals for Children Act (BPCA). While customised 3D-printed paediatric medicines may qualify for BPCA incentives, they will likely require long-term safety monitoring and robust post-market surveillance [6].

The shift toward 3D-printed paediatric medicines necessitates new regulatory standards that address decentralised production, novel excipients, GMP-compliant printer design, and the unique quality-control demands of personalised 3D-printed dosage forms.

## 8. Conclusions and Future Direction

The use of 3D printing in paediatric pharmaceutics marks a significant shift towards personalised, patient-centred treatment. Over the past three decades, since the first printed tablet prototypes were made in 1996, 3DP has evolved into a flexible manufacturing platform capable of creating complex geometries, customised release profiles, and accurate doses within minutes through computer-aided, layer-by-layer fabrication [120]. Its capacity to challenge the “one-size-fits-all” approach is especially important in paediatrics, where age-, weight-, and condition-specific dosing needs, as well as challenges related to swallowability and palatability, often restrict the effectiveness of traditional dosage forms.

Three-dimensional printing enables the creation of mini-tablets, orodispersible tablets and films, chewable forms, and soft or candy-like formulations that better align with children’s preferences and swallowing abilities [3,11,100]. In addition to increased acceptability, 3DP provides highly accurate dose titration, optimised release profiles, and the potential to simplify complex regimens in chronic diseases [6]. Semi-solid extrusion, binder jetting, and fused deposition modelling have all demonstrated the capacity to produce personalised paediatric dosage forms, including gel-based systems and rapidly disintegrating units [92]. These advantages position 3DP as a promising solution to long-standing limitations in both solid and liquid paediatric medicines—ranging from difficulty swallowing large tablets to stability issues and the use of potentially toxic solvents in oral liquids [104].

Despite this progress, the real-world application of 3DP in paediatric care remains limited by scarce clinical data, lack of dedicated regulatory pathways, and gaps in GMP standards for printers, software, excipients, and decentralised point-of-care production [6,14]. Standards for stability, batch definition, cleaning validation, and sterility remain developing areas, and healthcare systems must decide who is responsible for manufacturing and supervising personalised 3D-printed medicines [10]. Creating GMP-compliant 3D printers and pharmacopeial methods specific to 3D-printed dosage forms will be crucial to facilitating wider clinical adoption [11]. As regulators refine guidelines for point-of-care production and individualised dosing, the path toward safe clinical integration of 3DP into paediatrics is becoming increasingly evident [99].

Looking ahead, four-dimensional (4D) printing presents an exciting next step in the development of personalised drug delivery. Four-dimensional printing extends traditional 3D printing by incorporating time as a functional dimension, allowing printed structures to change their shape or properties in response to external stimuli such as temperature, pH, humidity, magnetic fields, or electrical signals [112]. In the field of pharmaceuticals, 4D printing is being explored for drug delivery systems capable of responding to environmental cues—such as pH, temperature, or humidity—to modulate structure, targeting, or drug release over time [110]. Meanwhile, 4D bioprinting combines living cells with biomaterials to create dynamic, tissue-like systems that imitate skin, bone, vasculature, or muscle, supporting both physiological modelling and controlled drug release [92]. Current progress largely relies on smart materials such as shape-memory polymers and shape-morphing hydrogels, although further optimisation in rheology, non-toxicity, and printability is required for clinical use.

In conclusion, 3D printing has already shown its potential to provide safer, more acceptable, and better-customised paediatric medicines—while 4D printing hints at a future where drug delivery systems dynamically adapt to a child’s physiology and environment. Ongoing research, targeted clinical trials, development of paediatric-appropriate excipients, and the creation of strong global regulatory frameworks will be vital for turning these innovations into standard therapeutic options. As personalised medicine becomes more central to healthcare, 3D (and 4D) printing technologies are ready to transform paediatric pharmacotherapy, offering treatments that are not only effective but also genuinely aligned with the unique needs and experiences of young patients.

## Figures and Tables

**Table 1:** Number of under-five deaths by cause in the years 2020 and 2021 (the latest data available on the WHO website as of 2025) [23].

Cause of Death	Number of Cases	
	2020	2021
Tetanus	8435	8132
HIV/AIDS	53,829	49,159
Measles	70,391	113,861
Meningitis/encephalitis	102,237	100,968
Tuberculosis	138,375	139,457
Sepsis and other infectious conditions of the newborn	175,858	172,221
Injuries	273,309	266,400
Congenital anomalies	425,062	407,926
Malaria	454,410	449,696
Diarrhoeal diseases	460,702	443,833
Birth asphyxia and birth trauma	604,332	588,181
Other Group 1 and Other noncommunicable (neonatal and under-5 only)	740,816	709,878
Acute lower respiratory infections	745,380	725,557
Prematurity	946,040	920,992

**Table 2:** Comparison of the three types of FDA’s Priority Review Voucher (PRV) programmes [29,30,31].

Points	Tropical Disease PRV	Rare Paediatric Disease PRV	Material Threat Medical Countermeasure PRV
Types of Drugs Eligible for PRV Awards	A drug intended to prevent or treat a tropical disease (e.g., tuberculosis, malaria, leprosy, etc.).	A drug intended to prevent or treat a rare disease or condition that is serious or life-threatening, and the serious or life-threatening manifestations must primarily affect individuals aged from birth to 18 years.	Drug intended to prevent or treat harm from a biological, chemical, radiological, or nuclear agent identified as a material threat (e.g., anthrax, botulism, smallpox, etc.).
Notice	Before 2014: 1 year After 2014: 90 days	90 days	At least 90 days
Fee (in 2020)	$2,167,116	$2,167,116	$2,167,116
Transferability	Transferable	Transferable	Transferable

**Table 3:** Biopharmaceutical properties of children in different age groups.

Pharmacokinetic Properties			Preterm Newborn Infants	Term Newborn Infants	Other Groups of Children
Absorption	Stomach	The bioavailability of the acid-labile compounds is better	Gastric pH is 6–8 at birth, but it reduces to 4 in the first 24 hours	Gastric pH is 6–8 at birth, and after a few hours, it reduces to 2–3	Gastric pH = 1–2
	Intestines	Motility is slow and irregular.	Absorption is prolonged; many APIs take longer to reach their maximum blood concentration.		
	Skin	Thinner stratum corneum, higher hydration of epidermis, weaker barrier function	Toxicity is more common.		
	Intramuscular injection	Less muscle tissue, worse blood flow, weaker muscle contractions	Unpredictable absorption		
	Rectal administration	Passive diffusion through membranes: lipophilic and less ionised molecules exhibit the best bioavailability	Differences between the age groups, also individually (depends on the rectal content, pH, surface of the rectal mucosa)		
Distribution	Body water ratio and extracellular body water	Hydrophile APIs have a larger volume to distribute	20% higher water volume than in adults		Higher extracellular body water volume and body water ratio than in adults
	Adipose tissue	Lipophile APIs have a smaller volume to distribute	Smaller adipose tissue volume than in adults		Time to time getting like the adult properties
	Plasma protein concentration	The level of protein-binding APIs is higher; therefore, their effect is stronger	Lower plasma protein concentration than in adults		Time to time getting like the adult properties
	Blood–brain barrier permeability	After birth, membrane permeability is higher; it decreases as children grow	Higher membrane permeability, which leads to CNS toxicity		Membrane permeability decreases and reaches the adult values
Metabolism	Enzyme concentration	The level of metabolising enzymes varies throughout the years	CYP3A7 enzyme is the most active one		Non-linear changing until adulthood
Elimination	GFR and tubular secretion	The elimination half-life of drugs being excreted by the kidneys is longer	GFR, tubular secretion is lower than in adults		Kidney function is complete by the age of 1 year

**Table 4:** Paediatric drug formulation administration characteristics.

Formulation Type	Description	Advantages	Disadvantages
Solid Oral Formulations	Tablets	Easy to administer Easy to produce Stable No dosage difficulties (one dose/piece)	Swallowing difficulties Taste masking is needed
	Mini tablets	Easy to administer Stable No dosage difficulties (one dose/piece)	Taste masking is needed Limited dose Difficult to formulate
	Hard capsules, soft gel capsules	Easy to administer Easy to produce Stable No dosage difficulties (one dose/piece)	Swallowing difficulties
	Granules	Easy to administer Easy to produce Stable No dosage difficulties (one dose/piece)	Taste masking is needed
	Pellets	Easy to administer Stable No dosage difficulties (one dose/piece)	Taste masking is needed Difficult to formulate
Chewable Formulations	Chewable tablets	Easy to administer (no choking) Stable No dosage difficulties (one dose/piece) Water not required	Taste masking is needed Difficult to formulate
	Soft chews	Easy to administer Stable No dosage difficulties (one dose/piece) Water not required	Taste masking is needed Difficult to formulate
	Chewing gums	Easy to administer Stable No dosage difficulties (one dose/piece) Water not required	Taste masking is needed Difficult to formulate
Orodispersible Formulations	Orodispersible tablets	Easy to administer (no choking) Fast disintegrating Stable No dosage difficulties (one dose/piece) Water not required	Limited dose Specialised packaging Difficult to formulate Patent protection
	Orodispersible films	Easy to administer (no choking) Fast disintegrating Stable Flexible dosage Water not required	Taste masking is needed Limited dose Specific API physicochemical properties Specialised packaging
	Orodispersible granules	Easy to administer (no choking) Fast disintegrating Stable Flexible dosage Water not required	Taste masking is needed Limited dose Specific API physicochemical properties Specialised packaging
	Orodispersible lyophilizates	Easy to administer (no choking) Fast disintegrating Stable Flexible dosage Water not required	Taste masking is needed Limited dose Specific API physicochemical properties Specialised packaging
	Orodispersible mini tablets	Easy to administer (no choking) Fast disintegrating Stable Flexible dosage Water not required	Taste masking is needed Limited dose Specific API physicochemical properties Specialised packaging
Liquid Formulations	Solutions	Easier to swallow Adjustable dosing	Stability issues Taste masking is required
	Emulsions	Easier to swallow Adjustable dosing	Stability issues Taste masking is required
	Suspensions	Easier to swallow Adjustable dosing	Stability issues Taste masking is required
	Infusions	Flexible dosage No swallowing difficulties	Needs healthcare professionals to administer Invasive (bad compliance)
	Injections	Flexible dosage No swallowing difficulties	Needs healthcare professionals to administer Invasive (bad compliance)
Special Delivery Systems	Inhaler Devices	Targeted delivery Improved bioavailability	Specific breathing techniques are required to be used properly Infants and toddlers cannot use it
	Transdermal Patches	Targeted delivery Improved bioavailability	Specific API physicochemical properties Limited dose
	Reservoir feeding bottle	Improve palatability Suitable for infants	Taste masking is needed Limited dose Specialised packaging Difficult to formulate
	Nipple Shield Delivery System	Improve palatability. Suitable for infants	Taste masking is needed Limited dose Specialised packaging Difficult to formulate
	Pacifiers	Improve palatability. Suitable for infants	Taste masking is needed Limited dose Specialised packaging Difficult to formulate
	Pulp Spoon with Single Dry Dose	Improve palatability. Suitable for infants	Taste masking is needed Limited dose Specialised packaging Difficult to formulate
	Solid Dosage Pen	Flexible dosage	Specialised packaging Difficult to formulate
	Straw	Easy to administer Flexible dosage	Specialised packaging Difficult to formulate Taste masking is needed

**Table 5:** General characteristics of the 3D printing technologies presented according to the ASTM categories.

	Material Extrusion (MEX)	Vat Photopolymerisation (VPP)	Powder Bed Fusion (PBF)	Material Jetting (MJT)	Binder Jetting (BJT)	Directed Energy Deposition (DED)	Sheet Lamination (SHL)
Pharma suitability	Very suitable	Emerging	Some potential	Good	Highly suitable	Not suitable	Very limited
Paediatric suitability	Most suitable	Moderately suitable	Moderately suitable	Most suitable	Most suitable	Not suitable	Not suitable
Physical state of the starting material	Semi-solid extrusion systems	Liquid/Resin systems	Powder-based systems	Liquid/Resin systems	Powder-based systems	Solid metal powder or wire	Semi-solid extrusion systems
Solidification/Bonding mechanism	Thermally induced fusion (melting)	Photopolymerisation	Thermally induced fusion (sintering/melting)	Photopolymerisation	Binder-mediated solidification	Thermally induced fusion (melting)	Mechanical or adhesive lamination
Advantages	Flexible doses, multilayer tablets, modified release, low cost	High resolution, complex geometries	Porous ODTs, precise internal structures	Precise dosing, films/ODTs, rapid printing	Fast dissolution ODTs, high API loading	—	—
Limitations	High temperatures (FDM), viscosity limits (SSE), API degradation risk	Photopolymer toxicity concerns, need for biocompatible resins	High heat, stability concerns, limited pharma-safe polymers	Viscosity restrictions, nozzle clogging	Requires special powders; limited to porous forms	High energy, metal- focused, incompatible with APIs	Mainly structural, not used for dosage forms
Comments	Enables mini-tablets, chewables, ODTs, gels; excellent for flexible dosing; compatible with child-friendly shapes and flavours.	High resolution; potential for soft gels or microstructures, but the safety of photopolymers remains a barrier.	Useful for porous structures/ODTs; limited by API degradation at higher temperatures.	Very precise dosing; ideal for ODFs, thin films, microdosing; suitable for infants and neonates.	Produces highly porous orodispersible tablets—ideal for children with swallowing difficulties; excellent disintegration times.	Metal-focused, high temperature, incompatible with APIs.	Structural manufacturing, not pharmaceutical.

## Data Availability

The original contributions presented in this study are included in the article. Further inquiries can be directed to the corresponding author.

## Abbreviations

The following abbreviations are used in this article:

3D	Three-Dimensional
3DP	Three-Dimensional Printing
4D	Four-Dimensional
ADME	Absorption, Distribution, Metabolism, Excretion
API	Active Pharmaceutical Ingredient
ASTM	American Society for Testing and Materials
AUC	Area Under the Curve
BJT	Binder Jetting
BPCA	Best Pharmaceutical for Children Act
CAD	Computer-Aided Design
CAT	Committee for Advanced Therapies
CHMP	Committee for Medicinal Products for Human Use
CLIP	Continuous Liquid Interface Production
COMP	Committee for Orphan Medicinal Products
CQAs	Critical Quality Attributes
CQPAs	Critical Quality and Performance Attributes
CVMP	Committee for Veterinary Medicinal Products
CYP	Cytochrome P450
DED	Directed Energy Deposition
DES	Deep Eutectic Solvents
DIW	Direct Ink Writing
DLP	Digital Light Processing
DoD	Drop-on-Demand
DPE	Direct Powder Extrusion
DPI	Dry Powder Inhalers
EC	Ethyl Cellulose
EHD	Electrohydrodynamic
E-Jet	Electrohydrodynamic Jet
EMA	European Medicines Agency
EXT	Semi-Solid Extrusion
FD&C	Food, Drug and Cosmetic Act
FDA	U.S. Food and Drug Administration
FDAMA	Food and Drug Administration Modernisation Act
FDASIA	Food and Drug Administration Safety and Innovation Act
FDM	Fused Deposition Modelling
FFF	Fused Filament Fabrication
GFR	Glomerular Filtration Rate
GMP	Good Manufacturing Practice
GST	Glutathione-S-Transferase
HME	Hot-Melt Extrusion
HMPC	Committee on Herbal Medicinal Products
HPC	Hydroxypropyl Cellulose
HPMC	Hydroxypropyl Methylcellulose
HPMCAS	Hydroxypropyl Methylcellulose Acetate Succinate
IJP	Inkjet printing
IM	Intramuscular
IND	Investigational New Drug
IP	Inkjet Printing
IV	Intravenous
MED	Melt Extrusion Deposition
MEW	Melt-Electrowriting
MEX	Material Extrusion
MJT	Material Jetting
NATs	N-acetyltransferases
NDA	New Drug Application
NIH	National Institutes of Health
NSDS	Nipple Shield Delivery System
ODFs	Orodispersible Films
ODTs	Orodispersible Tablets
PAM	Pressure-Assisted Microsyringe
PBF	Powder Bed Fusion
PBPK	Paediatric Physiologically Based Pharmacokinetic
PDCO	Paediatric Committee
PIP	Paediatric Investigation Plan
PK/PD	Pharmacokinetics and Pharmacodynamics
pMDI	Pressurised Metered-Dose Inhaler
pQTPP	Paediatric Quality Target Product Profile
PRAC	Pharmacovigilance Risk Assessment Committee
PREA	Paediatric Research Equity Act
PRV	Priority Review Voucher
PUMAs	Paediatric-Use Marketing Authorisations
PVP	Polyvinylpyrrolidone
QC	Quality Control
RACE	Research to Accelerate Cures and Equity for Children Act
SC	Subcutaneous
SCE	Screw Extrusion
SDP	Solid Dosage Pen
SE	Syringe Extrusion
SHL	Sheet Lamination
SLA	Stereolithography
SLM	Selective Laser Melting
SLS	Selective Laser Sintering
SMI	Soft Mist Inhalers
SSE	Semi-Solid Extrusion
STEP	Safety and Toxicity of Excipients for Paediatrics database
SULT	Sulfotransferase
TPMT	Thiopurine S-Methyltransferase
UDP	Uridine 5’-DiPhospho
UGTs	UDP-Glucuronosyl-Transferase
VPP	Vat Photopolymerisation
WHO	World Health Organisation
